# The Transforming Parasite *Theileria* Co-opts Host Cell Mitotic and Central Spindles to Persist in Continuously Dividing Cells

**DOI:** 10.1371/journal.pbio.1000499

**Published:** 2010-09-28

**Authors:** Conrad von Schubert, Gongda Xue, Jacqueline Schmuckli-Maurer, Kerry L. Woods, Erich A. Nigg, Dirk A. E. Dobbelaere

**Affiliations:** 1 Molecular Pathobiology, DCR-VPH, Vetsuisse Faculty, University of Bern, Bern, Switzerland; 2 Max-Planck Institute for Biochemistry, Martinsried, Germany; University of Vermont, United States of America

## Abstract

The protozoan parasite *Theileria* inhabits the host cell cytoplasm and possesses the unique capacity to transform the cells it infects, inducing continuous proliferation and protection against apoptosis. The transforming schizont is a multinucleated syncytium that resides free in the host cell cytoplasm and is strictly intracellular. To maintain transformation, it is crucial that this syncytium is divided over the two daughter cells at each host cell cytokinesis. This process was dissected using different cell cycle synchronization methods in combination with the targeted application of specific inhibitors. We found that *Theileria* schizonts associate with newly formed host cell microtubules that emanate from the spindle poles, positioning the parasite at the equatorial region of the mitotic cell where host cell chromosomes assemble during metaphase. During anaphase, the schizont interacts closely with host cell central spindle. As part of this process, the schizont recruits a host cell mitotic kinase, Polo-like kinase 1, and we established that parasite association with host cell central spindles requires Polo-like kinase 1 catalytic activity. Blocking the interaction between the schizont and astral as well as central spindle microtubules prevented parasite segregation between the daughter cells during cytokinesis. Our findings provide a striking example of how an intracellular eukaryotic pathogen that evolved ways to induce the uncontrolled proliferation of the cells it infects usurps the host cell mitotic machinery, including Polo-like kinase 1, one of the pivotal mitotic kinases, to ensure its own persistence and survival.

## Abstract 2

The transforming protozoan *Theileria* recruits Plk1, a host kinase that regulates mitosis, to its surface and engages spindle microtubules to secure its division and inheritance into both daughter cells.

## Author Summary

As part of their survival tactics, intracellular parasites often resort to cunning mechanisms to manipulate the cells they inhabit. *Theileria*, an important and particularly artful parasite of cattle in the tropics, transforms parasitized cells (that is, it induces continuous proliferation and protection from apoptosis—a state reminiscent of tumor cells). As a large, strictly intracellular syncytium, the transforming *Theileria* schizont cannot exit from the infected cell to invade other target cells. How then does the parasite ensure that each daughter cell, generated upon host cell division, remains infected and transformed? Our data show that the parasite co-opts the mitotic apparatus of the host cell and Plk1, a host protein kinase with a central regulatory role in mitosis and cytokinesis. As the host cell enters mitosis, the schizont binds to the microtubules that emanate symmetrically from the two spindle poles. This microtubule binding positions the schizont so that it spans the equatorial region of the mitotic cell where host cell chromosomes assemble. Then, as sister chromatids start to separate, the schizont associates with Plk1 and the central spindle that assembles between the separating chromosomes, with the activity of Plk1 presumably coordinating progression through mitosis with proper schizont positioning. This alignment with the central spindle positions the schizont to be included in the plane of cell division at the onset of cytokinesis, thus ensuring faithful passage of a *Theileria* schizont on to each daughter cell.

## Introduction

The apicomplexan parasites *Theileria annulata* and *T. parva* are transmitted by ticks and cause severe lymphoproliferative disease in cattle in large areas of Africa, the Middle East, and Asia. The pronounced pathology and high mortality are linked to the ability of *Theileria* to stimulate the uncontrolled proliferation of the cells it infects, inducing a phenotype typical of tumor cells. *T. parva* infects predominantly T- and B-lymphocytes, whereas *T. annulata* targets B-lymphocytes and macrophages/monocytes. *Theileria*-transformed cells proliferate independently of antigenic stimulation or exogenous growth factors. Parasitized cells become resistant to apoptosis [1]–[3] and acquire the capacity to invade and multiply in non-lymphoid as well as lymphoid tissues (reviewed in [4]–[6]). In buffalo, the natural host of *Theileria*, and in domestic animals that survive infection, the parasite persists for years, resulting in a carrier state.

*Theileria* parasites differ from other Apicomplexan parasites, such as *Plasmodium* and *Toxoplasma*, in that they do not reside in a parasitophorous vacuole. Shortly after entry, the invading sporozoite dissolves the surrounding host cell membrane. Free in the cytosol, the sporozoite immediately associates with host cell microtubules (MTs) and differentiates into the schizont stage of the life cycle [7], a multinucleated syncytium that maintains the host cell in a transformed state.

Significant progress has been made understanding how this parasite manipulates its host. To achieve transformation, the parasite induces the activation of host cell signaling pathways that control cell proliferation and survival. This includes signaling pathways that regulate G1 to S transition, resulting in host cell DNA replication [6],[8]. Transformation is entirely dependent on the presence of the parasite in the cytoplasm, however, and upon killing of the parasite by treatment with a specific theilericidal drug, cells lose the transformed phenotype, stop proliferating [9], and reacquire sensitivity to apoptosis.

*Theileria*-infected cells can be cultured indefinitely in vitro, and in established cultures, more than 95% of the cells harbor the parasite. Parasite and host cell DNA replication is asynchronous, with the schizont predominantly undergoing DNA synthesis and nuclear division as the host cell enters mitosis [10]. Schizonts are strictly intracellular and, to maintain the host cell transformed phenotype, the organism must be passed on to both daughter cells each time the host cell goes through mitosis and cytokinesis (M phase). Viruses that transform their host cells have evolved mechanisms to guarantee their persistence in proliferating cells. In the case of retroviruses, this involves integration into the host cell genome (reviewed in [11]). DNA viruses such as Kaposi's sarcoma-associated herpesvirus, Epstein-Barr virus, or papillomavirus have evolved a conserved strategy to ensure genome segregation during mitotic division that involves tethering episomal viral genomes to mitotic chromosomes using virus-encoded proteins (reviewed in [12]). How can *Theileria*, as a large and complex eukaryotic syncytium, persist in a continuously dividing host cell? Early microscopic observations have provided first clues for an involvement of the host cell mitotic apparatus [13]. However, the kinetics and molecular mechanism underlying the interaction between the schizont and its host cell during mitosis have not yet been investigated in detail.

The regulation of mitosis and cytokinesis involves a range of mitotic kinases, motor proteins, and MT structures that undergo extensive reorganization to coordinate diverse functions such as chromosome segregation and cell division. In line with their multiple functions, regulatory proteins are subject to extensive spatio-temporal regulation, translocating between different structures where they fulfill specific functions. The mitotic spindle, which forms during early mitosis, consists of astral and interpolar MTs as well as kinetochore fibers that attach to condensed chromosomes. Cells are only allowed to exit mitosis when all chromosomes are correctly aligned on the mitotic spindle. At this point, Cdk1/cyclin B is inactivated, and during the ensuing anaphase, kinetochore MTs shorten to deliver the sister chromatids towards the poles. In the zone between the separating sets of sister chromatids (spindle midzone), a specialized structure, the central spindle, forms, consisting of bundles of antiparallel MTs with overlapping plus ends. By recruiting a specific set of regulatory proteins, including mitotic kinases such as Polo-like kinase 1 (Plk1) and Aurora B, as well as activators of the RhoA GTPase, the central spindle provides an important signaling platform that determines the plane of cleavage furrow formation and cytokinesis. For detailed information on central spindle assembly and cytokinesis, the reader is referred to recent reviews [14]–[16].

Plk1 has a broad range of functions during different stages of cell division and is subject to complex spatial and temporal control (reviewed in [17]–[19]). Plk1 is degraded at the end of M phase and protein levels remain low during G1. Levels increase when cells enter S phase, accumulating strongly during G2. As the cell prepares for mitosis, Plk1 can be found localized to centrosomes and a first accumulation at centromeres can be observed. Upon progression through prometaphase and metaphase, Plk1 associates with spindle poles and kinetochores. The choice of Plk1 docking partners is regulated by Cdk1 [20], and upon Cdk1 inactivation at anaphase, Plk1 is released from kinetochores and recruited to the newly forming central spindle. Finally, during cytokinesis, Plk1 is found localized to the midbody. By phosphorylating different interacting partners, Plk1 contributes to a number of events linked to cytokinesis such as contractile ring formation and cleavage furrow ingression [17],[21]–[23]. The application of RNAi-mediated Plk1 knock-down or Plk1 inhibition using dominant negative mutants proved highly valuable to dissect the early functions of Plk1 in mitosis. However, important new insights into the role of Plk1 during cytokinesis only became possible with the recent development of specific chemical tools that allow the rapid and complete inactivation of Plk1 at precise time-points during mitosis, and without interfering with earlier functions [24]–[27]. Armed with these new tools, we analyzed how the *Theileria* schizont interacts first with the mitotic spindle and subsequently with the central spindle during host cell M phase. We show that the parasite establishes a close interaction with both structures and found that its association with the central spindle depends on catalytically active Plk1. The latter associates with the schizont surface in a biphasic manner and recruitment is negatively regulated by host cell Cdk1.

## Results

### The *Theileria* Schizont Interacts with De Novo Synthesized Astral and Spindle Midzone MTs

**Figure 1:**
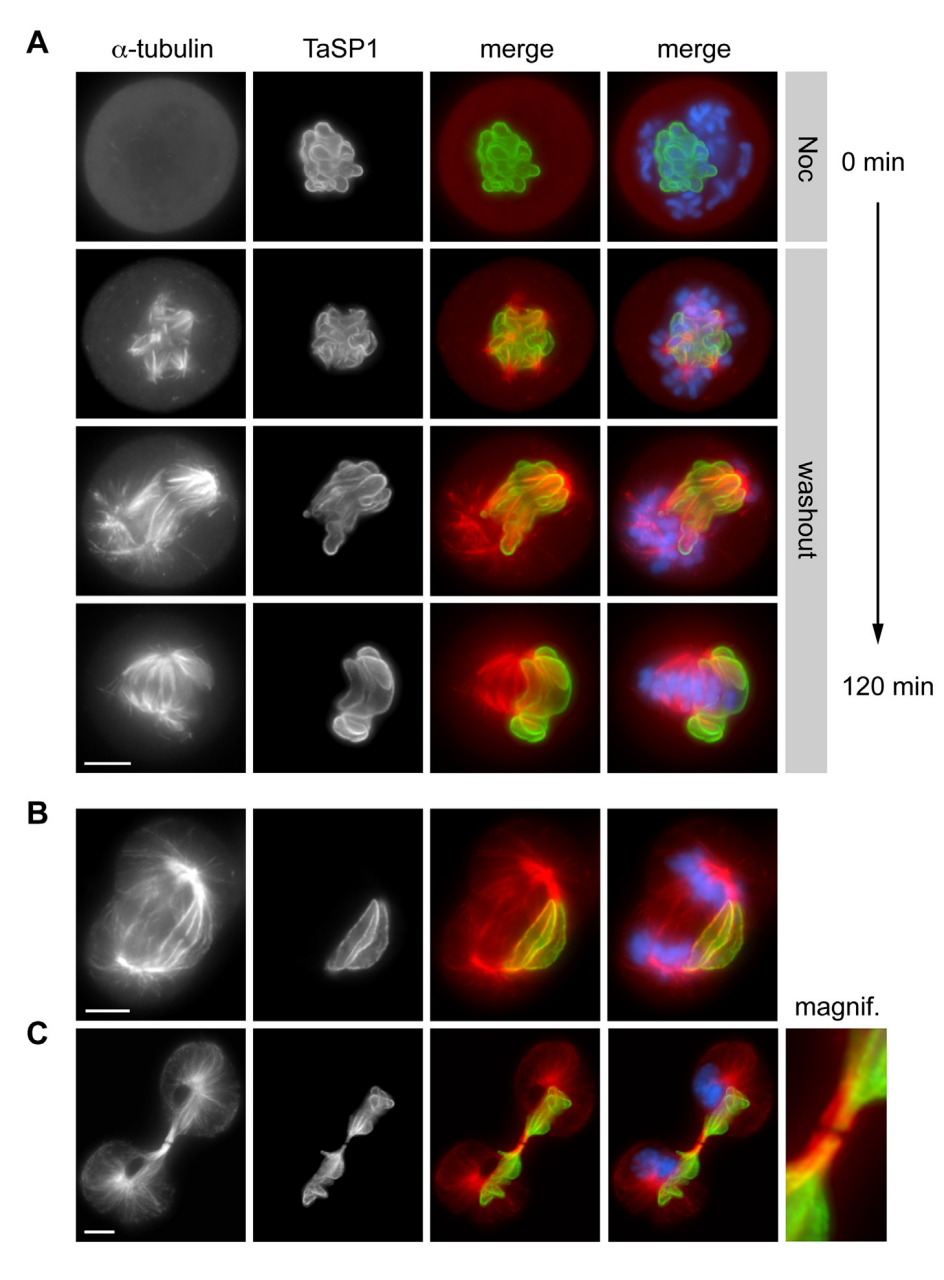
*Theileria* schizont interaction with mitotic and spindle midzone MTs. (A) *T. annulata*-transformed cells were arrested in prometaphase by treatment with nocodazole (Noc; top panels). Upon removal of nocodazole, MT polymerization was monitored by immunofluorescence microscopy (IFM) for up to 120 min. Cells were stained with antibodies directed against the schizont surface marker TaSP1 and α-tubulin; DNA was stained with DAPI; “merge” represents an overlay of the images. Noc, nocodazole; “washout” indicates removal of nocodazole. (B, C) Unsynchronized *T. annulata*-transformed cells undergoing anaphase and telophase/cytokinesis. The panel labeled “magnif” represents a magnification of the midbody region of the dividing cell shown in (C). Scale bars represent 5 µm.

To monitor the interaction of the schizont with de novo synthesized MTs, *T. annulata*-transformed cells were exposed to nocodazole, a drug that inhibits MT polymerization. After 16 h of treatment, mitotic cells lacking MTs were arrested in prometaphase because of spindle checkpoint activation. Within minutes of nocodazole removal, new bundles of MTs formed that aligned closely with the parasite surface, stained with anti-TaSP1, a commonly used schizont surface marker (Figure 1A) [28]. The appearance of multiple small MT asters early upon nocodazole release is most likely not due to parasite-induced MT nucleation as such asters could also be observed in uninfected bovine control cells (Figure S1A). At metaphase, the parasite was found oriented symmetrically towards both spindle poles, straddling the chromosomes assembled in the metaphase plate. At anaphase, spindle midzone MTs, located between the separating sister chromatids, were aligned longitudinally along large sections of the parasite as dense MT bundles (Figure 1B). As the cleavage furrow ingressed, central spindle MTs, including those associated with the parasite, became compacted and, during cytokinesis, both chromosome sets and the parasite were equally distributed between the two daughter cells (Figure 1C). TaC12 cells are of macrophage origin and in order to allow a morphological comparison, different stages of mitosis/cytokinesis as observed in a bovine control cell line of macrophage origin (BoMac) or cells that no longer contain the parasite are presented in Figure S1B and C.

The accumulation of host cell MT bundles at the schizont surface does not require bipolar spindles as it could also be observed in cells treated with monastrol, a small-molecule inhibitor of the mitotic kinesin Eg5 that induces the formation of monopolar half-spindles (Figure S2) [29]. In monastrol-treated cells the parasite is less mobile compared to untreated cells, facilitating live imaging of MT interactions with the parasite surface. A kymograph analysis suggested that host cell astral MT bundles appear to be stably associated with the schizont surface (Figure S2).

### Biphasic Cell-Cycle Dependent Recruitment of Host Plk1 to the Schizont Surface

**Figure 2:**
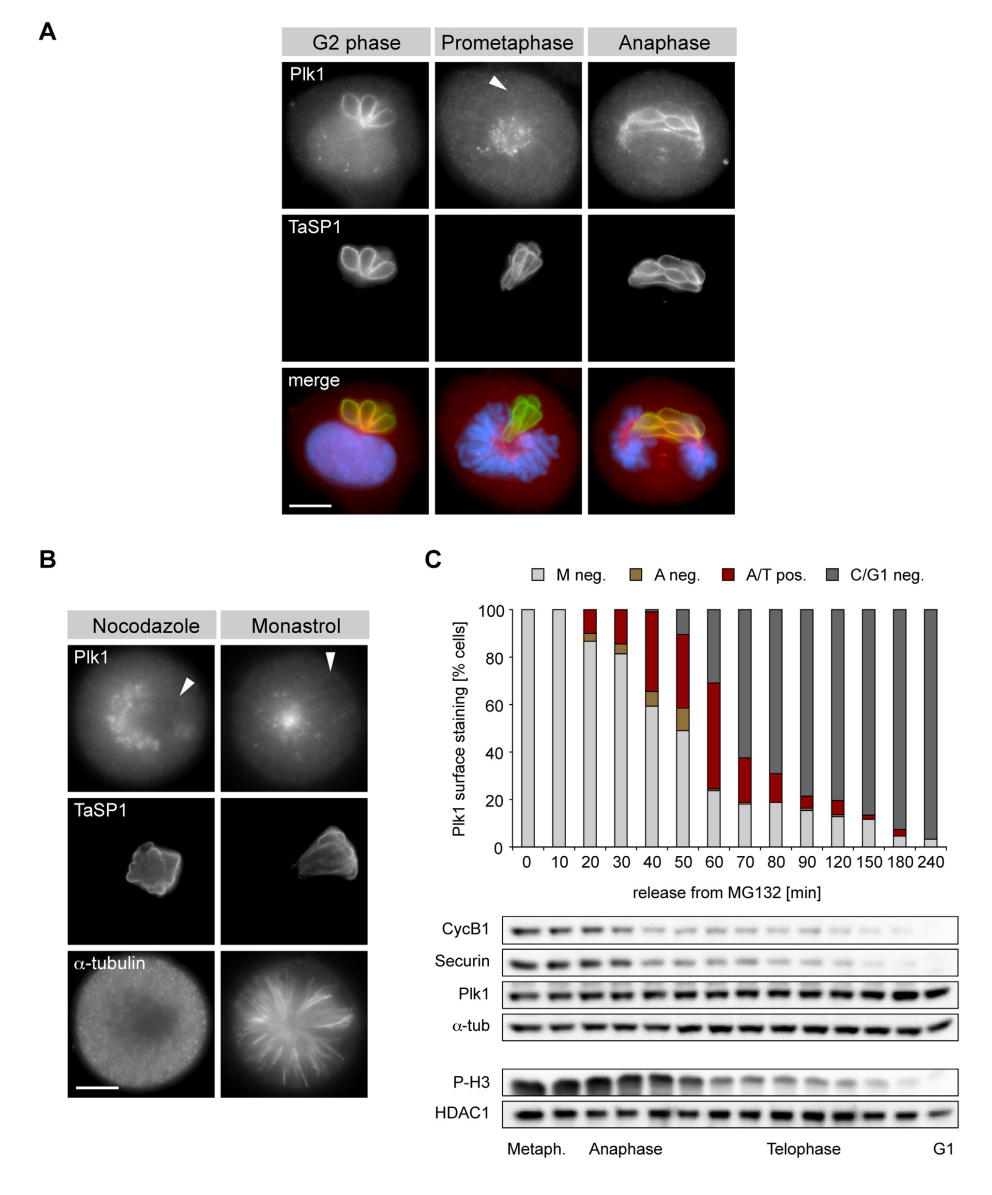
Biphasic recruitment of host Plk1 to the schizont surface. (A) *T. annulata*-transformed cells were stained with anti-Plk1, anti-TaSP1, and DAPI and analyzed by IFM. Cell cycle stages are indicated. Arrowhead indicates the position of the parasite. (B) Cells arrested in prometaphase by treatment with nocodazole (left panels) or monastrol (right panels) and analyzed by IFM using antibodies to Plk1, TaSP1 and α-tubulin. Arrowheads indicate the position of the parasite. (C) Cells were released from MG132-mediated metaphase arrest and the percentage of cells containing parasites with surface-bound Plk1 was determined by IFM (*n*≥100 cells/sample). M neg.: metaphase cells, parasite Plk1-negative; A neg.: anaphase cells, parasite Plk1-negatve; A/T pos.: Ana- & Telophase cells, parasite Plk1-positive; C/G1 neg.: cells in cytokinesis or G1, parasite Plk1-negative. Progression through M phase was monitored by immunoblot analysis using antibodies as indicated. HDAC1 (histone deacetylase 1) and α-tubulin were used as loading controls. Scale bars represent 5 µm.

In previous work, we demonstrated that *T. parva* and *T. annulata* can aggregate the host cell kinases IKK1 and IKK2 at its surface, activating a signaling pathway that promotes survival of the transformed host cell [30]. Using immunofluorescence microscopy, we investigated whether this might also apply to mitotic kinases. In unsynchronized cultures of *T. annulata*-transformed cells, Plk1 was found to localize to the surface of the schizont in approximately 30% of cells. This was most striking in cells in G2 and also in cells undergoing anaphase (Figure 2A). Intriguingly, during prometaphase and metaphase, Plk1 was consistently absent from the schizont surface. The association of Plk1 with the parasite during different phases of the cell cycle is presented in Figure S3. Plk1 was not detectable in cells in G1. During G2, when Plk1 is abundantly expressed, prominent labeling of the schizont surface could be observed, coinciding with the time at which Plk1 started to accumulate at host cell centromeres. Binding to the schizont was maintained until nuclear accumulation of cyclin B1 and nuclear envelope breakdown became apparent during prophase (unpublished data). Once cells reached prometaphase and metaphase, Plk1 localized predominantly to the spindle poles and kinetochores but was not associated with the schizont. With the onset of anaphase, Plk1 re-accumulated on the parasite surface. In cells progressing to telophase, Plk1 association with the parasite was largely restricted to the section of the schizont that is incorporated into the central spindle.

To analyze Plk1 recruitment to the parasite surface in more detail, we used different synchronization protocols. The different synchronization strategies used in this study are depicted schematically in Figure S4A. In a first set of experiments, *Theileria*-infected cells were synchronized in early S phase using a thymidine block. Cells were released from the arrest and Plk1 association with the schizont monitored by immunofluorescence microscopy as cells progressed towards G2 and M phase (Figure S5). The percentage of cells containing Plk1-binding schizonts increased progressively as they advanced through G2 and then decreased as they entered mitosis. Progression into M phase was monitored by immunoblot analysis of lysates, prepared at each time point, using antibodies specific for phospho-histone H3. Immunoblot analysis also showed that reduced association of Plk1 with the parasite as cells proceeded into mitosis was not due to declining Plk1 levels as these continued to increase during the course of the experiment. In cells synchronized in prometaphase by treatment with nocodazole, Plk1 could not be detected on the parasite (Figure 2B, left panels) confirming our observation made in unsynchronized cultures. The lack of Plk1 binding could not be attributed to the lack of MTs as identical observations were made in cells synchronized in prometaphase by treatment with monastrol (Figure 2B, right panels).

We next defined at which stage after metaphase the Plk1-parasite interaction was reinstated. By blocking the degradation of cyclin B using the proteasomal inhibitor MG132, inactivation of the Cdk1/cyclin B complex can be prevented, thus synchronizing cells in a metaphase-like state (see scheme Figure S4A) [26]. To follow progression through anaphase, telophase and exit from M phase, the degradation of cyclin B1 and securin, and the disappearance of the phospho-histone H3 epitope was monitored by immunoblot. Consistent with earlier observations, no parasite-associated Plk1 could be detected in metaphase-arrested cells (Figure 2C). Upon release from metaphase arrest, Plk1 associated with the parasite surface as soon as anaphase started. Binding was lost as cells completed cytokinesis and entered interphase/G1.

The capacity to induce transformation of the mammalian host cell is restricted to the schizont stage of *Theileria* and host cell proliferation ceases when the schizont differentiates to the next life cycle stage in a process called merogony [31],[32]. Figure S4B provides an overview of the mammalian stages of the *Theileria* life cycle. Merogony can also be induced in vitro by exposure to heat shock [31] or treatment with chloramphenicol [33]. Merogony is an asynchronous stochastic process that occurs in individual cells over a period of 4 to 10 d. Upon induction, the number of cells harboring parasites expressing the differentiation marker TamR1 gradually increased, reaching up to 45%. This was accompanied by a pronounced reduction in the number of cells expressing Plk1, including cells containing the transforming schizont with Plk1 located to its surface (from typically 30% in normal cultures to <10%). The reduction in the number of cells expressing Plk1 likely reflects cell cycle arrest in G1 or G0. As parasites proceeded into merogony, they lost the capacity to bind Plk1 (Figure S6). In 13% of the cells, scattered, but weak, Plk1 binding to parasites in early stages of differentiation could still be observed (an example is shown in Figure S6, middle row). No Plk1 binding could be detected when parasites had completed differentiation.

Taken together, these data show that host cell Plk1 interacts with the surface of the parasite in a biphasic manner and that this is restricted to the transforming stage of the life cycle.

### Plk1 Binding to the Schizont Surface Is Modulated by Cdk1 and Does Not Require Plk1 Catalytic Activity

The pattern of Plk1 binding to the schizont surface correlated inversely with the spectrum of Cdk1/cyclin B activity. While Cdk1-mediated phosphorylation can create docking sites for Plk1 [34],[35], in other cases Cdk1 prevents binding. Plk1 can also “self-prime”, however, and the choice of Plk1 docking partners through the course of mitosis and cytokinesis is thus controlled by the activation state of Cdk1 and that of Plk1 itself [20],[36],[37].

**Figure 3:**
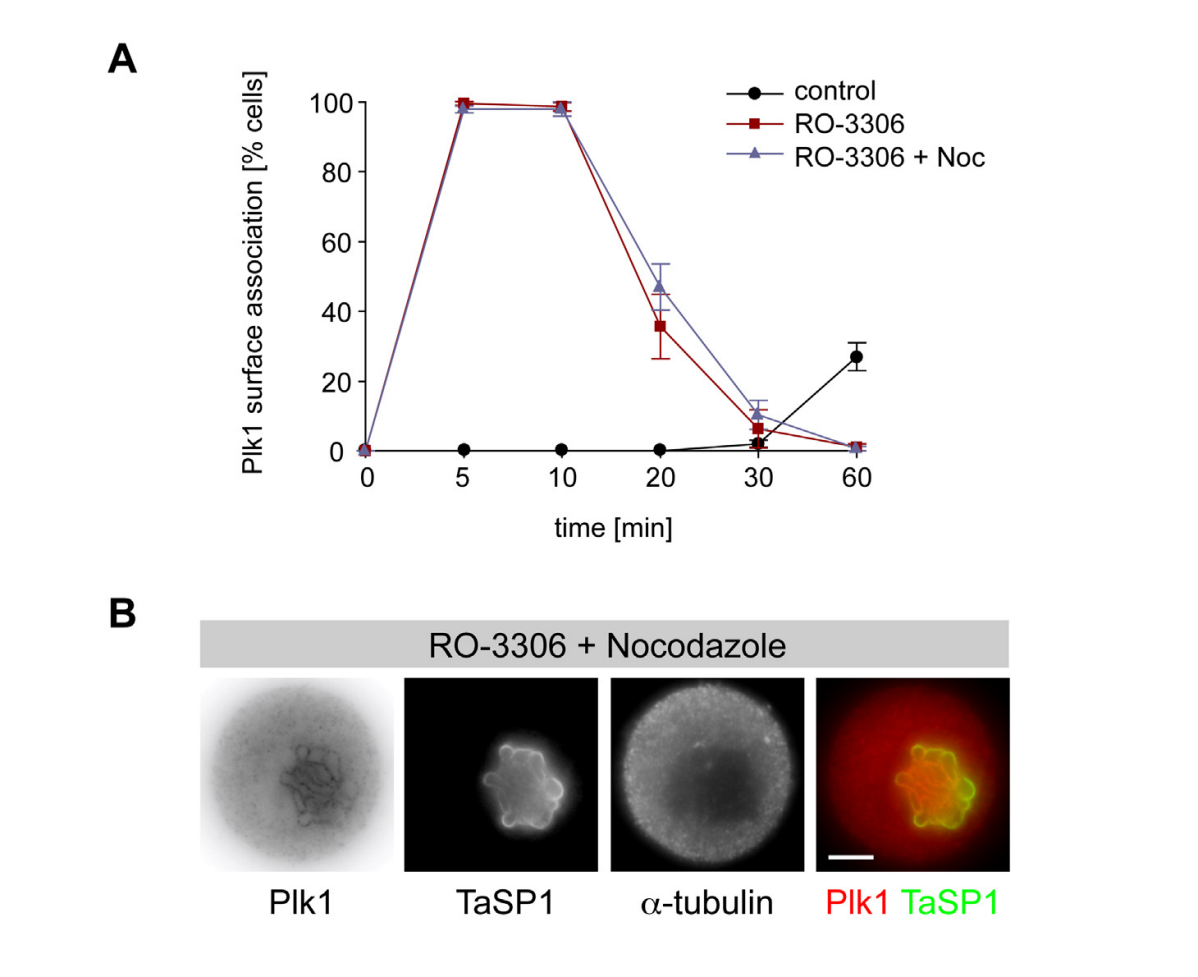
Cdk1 modulates Plk1 binding to the schizont surface. Cells arrested in prometaphase by nocodazole treatment were washed and immediately exposed to the Cdk1 inhibitor RO-3306, and Plk1 binding to the schizont surface was analyzed by IFM using antibodies to Plk1 (inverse gray/red), anti-TaSP1 (green), and anti-α-tubulin. (A) Time-course analysis showing the percentage of cells harboring parasites with surface-bound Plk1 following treatment with Cdk1 inhibitor in the presence (RO-3306 + Noc) or absence (RO-3306) of nocodazole. Control cells were released from nocodazole block in the presence of DMSO (control). Data represent the mean of three experiments (*n* = 150 cells/sample), and error bars indicate standard deviation (SD). (B) Cells treated with the Cdk1 inhibitor RO-3306 in the presence of nocodazole (10 min). Scale bars represent 5 µm.

Cdk1 activity is required to maintain the mitotic state. Unscheduled inactivation of Cdk1 during mitosis induces a cytokinesis-like process that takes place before chromosome alignment and proper chromatid segregation has occurred (Figure S4A) [38]. As shown in Figure 2B, Plk1 is not associated with the schizont in *Theileria*-transformed cells synchronized in prometaphase. Blocking Cdk1 activity by treatment with the chemical inhibitor RO-3306, however, induced the immediate accumulation of Plk1 on the schizont surface (Figure 3A and B). This also occurred in the presence of nocodazole, indicating that Plk1 recruitment to the parasite surface does not require mitotic MTs. In both cases, the association induced by Cdk1 inhibition was transient and downregulated within 30 min.

**Figure 4:**
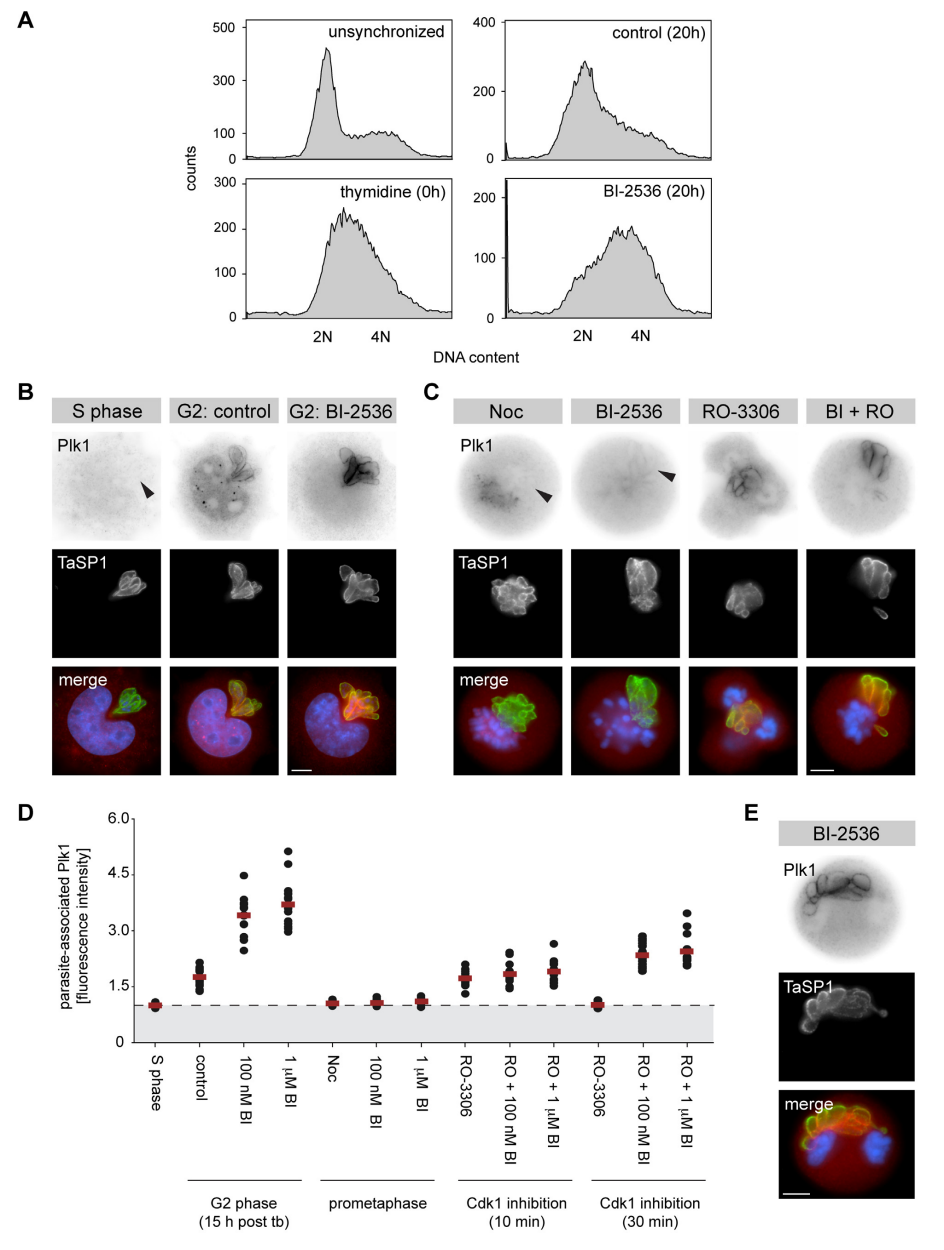
Plk1 binding to the parasite surface does not require Plk1 catalytic activity. (A) Unsynchronized or S phase-synchronized cultures of *T. annulata*-infected cells were analyzed by flow cytometry (left panels). Synchronized cultures (thymidine) were released for 20 h with either DMSO (control) or 100 nM BI-2536 (BI-2536). 2N, G1 phase; 4N, G2/M phase. (B) Representative micrographs of TaC12 cells in S phase arrest (S phase) or G2 phase cells that were released from S phase for 15 h in the presence of DMSO (control) or 1 µM BI-2536 (BI-2536). Arrowhead indicates the position of the parasite. Cells were stained for Plk1 and TaSP1 and analyzed by IFM; DNA was stained with DAPI. (C) S phase-synchronized TaC12 cells were released in the presence of nocodazole (Noc) or 1 µM BI-2536 (BI-2536), and prometaphase-arrested cells were harvested after 15 h. Nocodazole-blocked prometaphase cells were washed and released with Cdk1 inhibitor (RO-3306); cells that arrested in prometaphase by BI-2536 treatment were additionally treated with Cdk1 inhibitor (BI + RO). Cells were analyzed by IFM after 10 min incubation with Cdk1 inhibitor. Arrowheads indicate the position of the parasite. (D) The fluorescence intensity of Plk1 staining at the parasite surface and in the host cell cytoplasm was measured at multiple locations in TaC12 cells; each dot represents the ratio of the average parasite surface and cytoplasmic Plk1 level of an individual cell. The following cells were analyzed: cells arrested in S phase by thymidine treatment (S phase), G2 phase cells released for 15 h from thymidine block in the presence of DMSO (control) or BI-2536 (100 nM BI, 1 µM BI), and cells that arrested in prometaphase upon release from thymidine block in the presence of nocodazole (Noc) or BI-2536 (100 nM BI, 1 µM BI); nocodazole was removed by washing and arrested cells subsequently treated with Cdk1 inhibitor alone (RO-3306) or Cdk1 and Plk1 inhibitors (RO +100 nM BI, RO +1 µM BI) for 10 or 30 min. Fifteen cells were analyzed per sample and time point; horizontal red lines represent the average fluorescence intensity of each population; dashed line indicates a ratio of 1.0; tb, thymidine block. (E) Cells synchronously released from MG132-induced metaphase arrest were treated with 1 µM BI-2536, 15 min upon release, and analyzed by IFM. Scale bars represent 5 µm.

As Plk1 binding to substrates can result from self-priming [20],[36], we investigated the requirement of Plk1 catalytic activity for Plk1 binding to the parasite surface in more detail. TaC12 cells synchronized in S-phase were released from thymidine block and cultured in the presence or absence of the specific Plk1 inhibitor BI-2536 [39],[40] at concentrations from 100 nM up to 1 µM. In agreement with the described role of Plk1 in early mitosis [27],[39]–[42], cells released from S-phase in the presence of BI-2536 accumulated in G2/M and underwent prometaphase-like mitotic arrest, whereas control cells progressed through mitosis into G1 (shown for BI-2536 at 100 nM; Figure 4A). Accumulation in prometaphase upon BI-2536 treatment was also observed in unsynchronized TaC12 cultures (Figure S7A). In BI-2536 cells that were still in G2, Plk1 was readily detected at the parasite surface, and this was even more marked at higher doses of BI-2536 (Figure 4B). In agreement with our observations described above, Plk1 association with the parasite was strongly reduced in those cells that had arrested in “prometaphase” (Figure 4C and D). When Cdk1 was inhibited in cells arrested in “prometaphase,” Plk1 immediately reaccumulated at the parasite surface; this also occurred when BI-2536 was added at concentrations as high as 1 µM (Figure 4C and D). In the presence of BI-2536, Plk1 interaction with the parasite could still be detected in the majority of the cells after 30 min, whereas Plk1 was absent from the parasite in cells treated only with Cdk1 inhibitor at that time point (Figure 4C). Importantly, in the presence of BI-2536, the pronounced ectopic cleavage furrow formation observed upon Cdk1 inhibition was inhibited (Figure S7F), confirming the reported role for Plk1 in regulating furrow ingression (see also below and Figure S12) [21],[22],[24]–[27].

We also determined whether Plk1 recruitment to the parasite during normal anaphase required Plk1 activity. In the presence of BI-2536, cells released from metaphase arrest entered anaphase but failed to undergo furrow ingression. In these cells, Plk1 was found associated with the parasite surface (Figure 4E).

In further control experiments, BI-2536 was found to exert the same inhibitory effects in TaC12 cells as described in other systems; this included failure to maintain bipolar spindles (Figure S7B and C), cytokinetic failure (not shown), and the accumulation of binucleate cells (Figure S7D and E).

Although residual Plk1 activity can never be completely excluded, our findings indicate that Plk1 docking to the parasite surface can occur in the presence of Plk1 inhibitor concentrations that potently block several physiological functions of Plk1 in the cell. Together, these data indicate that Cdk1 negatively regulates Plk1 association with the schizont surface and that Plk1 binding does not appear to require catalytic activity.

### Plk1 Binds to the *Theileria* Schizont via Its Polo-Box Domain

**Figure 5:**
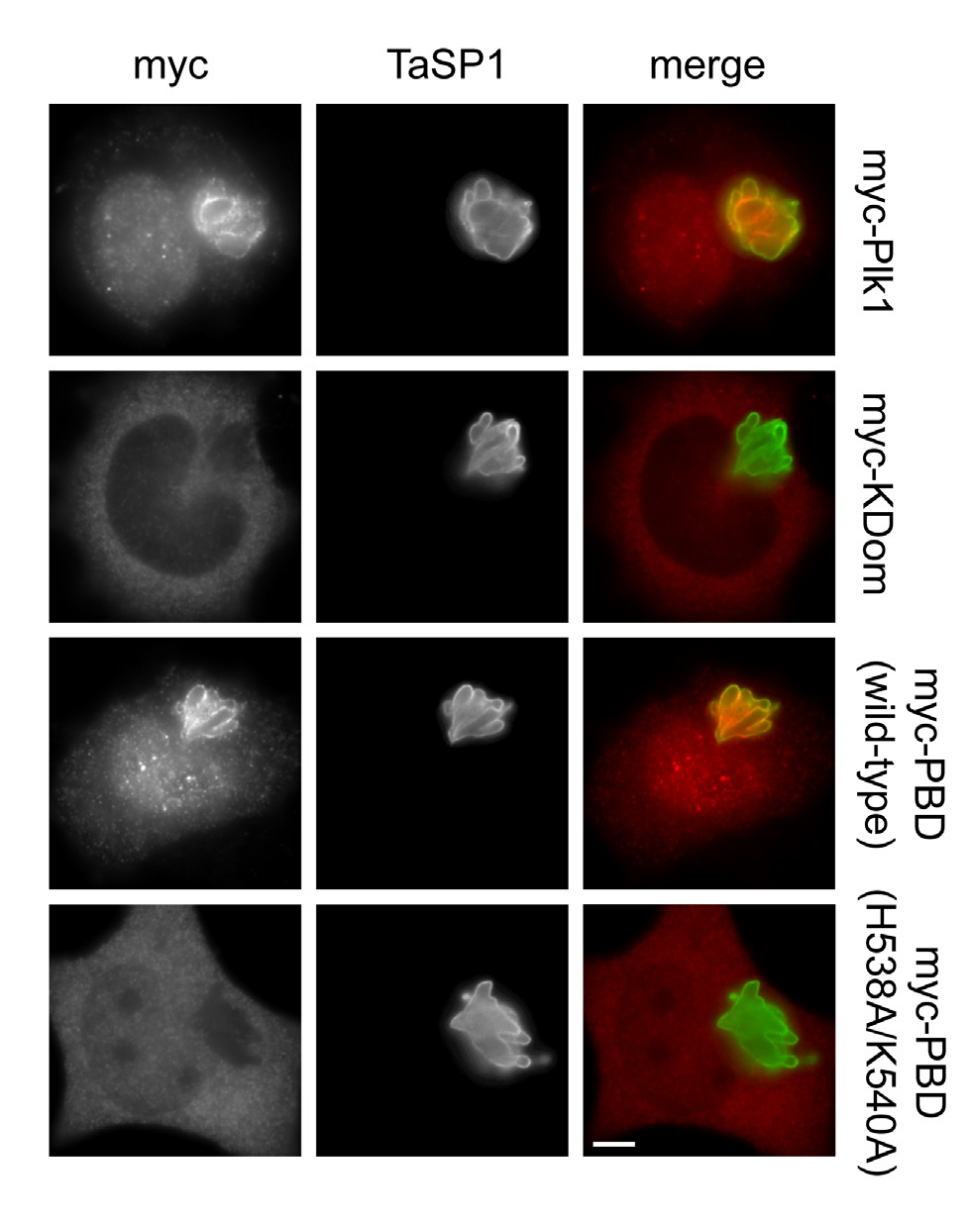
Plk1 binds to the *Theileria* schizont via its polo-box domain. *T. annulata*-infected cells were transfected with plasmids encoding myc-tagged versions of full-length Plk1 (myc-Plk1), Plk1 kinase domain (myc-KDom), Polo-box domain (myc-PBD, wild type), or H538A/K540A mutant PBD (myc-PBD H538A/K540A) and analyzed by IFM using anti-myc and anti-TaSP1 antibodies. Scale bar represents 5 µm.

**Figure 6:**
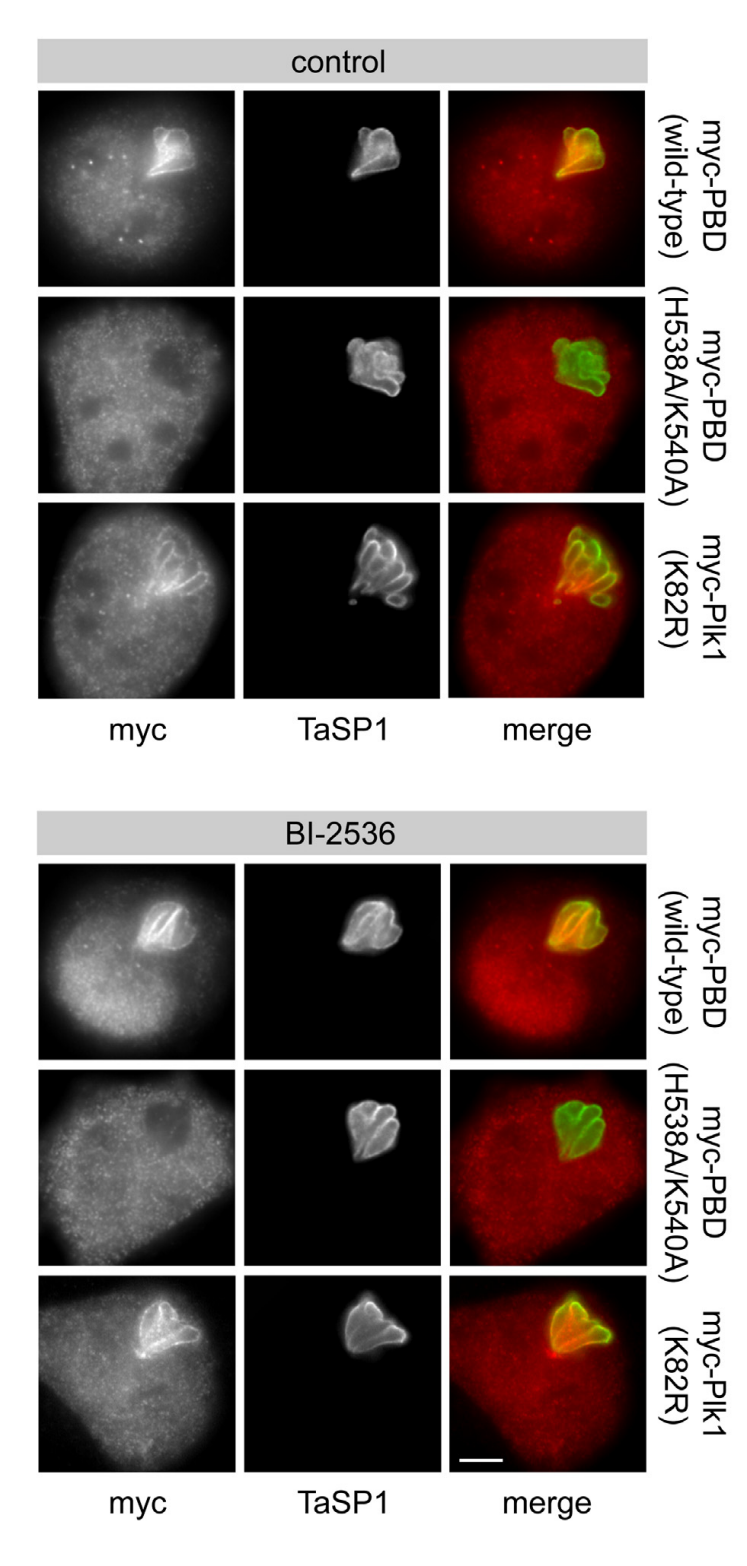
The binding of ectopically expressed Plk1 to the parasite surface does not require Plk1 catalytic activity. *T. annulata*-infected cells were transfected with plasmids encoding myc-tagged versions of Polo-box domain (myc-PBD, wild type), H538A/K540A mutant PBD (myc-PBD H538A/K540A), or catalytically inactive Plk1 (myc-Plk1 K82R) and cultured in the presence or absence of BI-2536 as indicated. Cells were analyzed by IFM using anti-myc and anti-TaSP1 antibodies. Scale bar represents 5 µm.

To determine which region of Plk1 is responsible for binding to the schizont surface, different myc-tagged forms of Plk1 were transiently expressed in *T. annulata*-transformed cells. Immunofluorescence analysis showed that myc-tagged Plk1 localized to the parasite surface (Figure 5). When the N-terminal kinase domain (KDom) and the C-terminal Polo-box domain (PBD) were expressed separately, only the latter showed binding. H538 and K540 are important residues in the PBD that are required for phospho-ligand binding [35],[43]. Mutation of these residues to alanine completely abrogated PBD binding to the parasite, indicating that a functional PBD with an intact phosphopeptide recognition domain is required for Plk1 interaction with the parasite. To exclude the potential participation of endogenous Plk1 in the creation of PBD docking sites on the parasite surface, cells transfected with PBD constructs were treated with BI-2536 (Figure 6). Inhibition of Plk1 activity did not interfere with myc-PBD, myc-PBD^H538A/K540A^, or kinase dead full-length Plk1 (myc-Plk1^K82R^) binding. This was also observed when doses as high as 1 µM were used (Figure S8), confirming that recruitment is, in all likelihood, independent of Plk1 activity.

### The Schizont Recruits Host Cell Central Spindles to Its Surface in a Plk1-Dependent Manner

**Figure 7:**
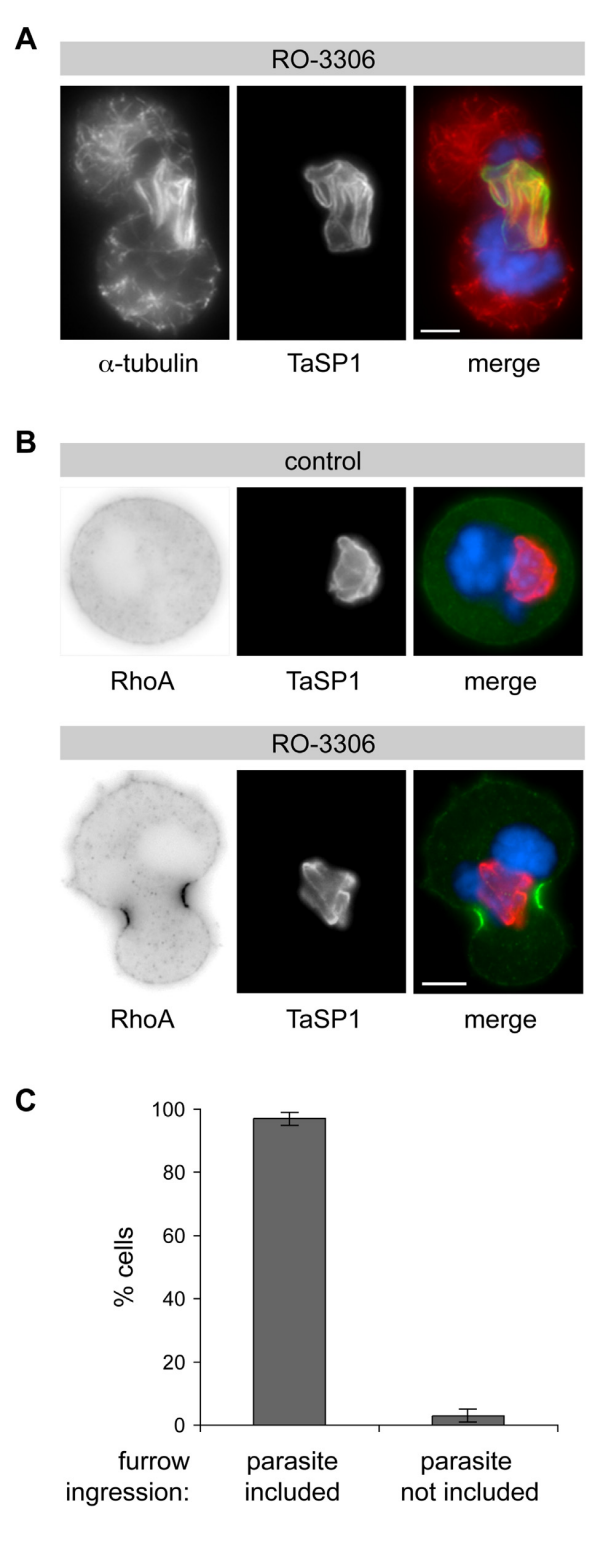
Upon induction of precocious cell division, cleavage furrows form in the immediate vicinity of the parasite. (A) *T. annulata*-transformed cells were synchronized in prometaphase by treatment with nocodazole. Upon nocodazole washout, precocious cell division was induced using RO-3306. After 20 min of treatment, newly formed microtubules were visualized by α-tubulin staining and the parasite was detected by anti-TaSP1. (B) RO-3306-induced RhoA accumulation and cleavage furrow formation are influenced by the position of the schizont. Cells synchronized in prometaphase as in (A) were treated for 20 min with either RO-3306 or solvent only (control) upon nocodazole washout. (C) The position of the parasite in cells undergoing cleavage furrow formation was monitored by IFM 30 min after RO-3306 induction shown in (A) and (B). Bars represent the percentage of cells in which the schizont was included in the cleavage furrow. Data represent the mean of three experiments with *n* = 60 cells/sample; error bars indicate SD. Scale bars represent 5 µm, and DNA was stained with DAPI.

Plk1 is closely involved in central spindle function and in helping to determine the site of contractile ring assembly and furrow ingression, ultimately resulting in cytokinesis (see reviews [14],[15] and references therein). To test to what extent the schizont is linked to these processes, precocious anaphase and cytokinesis-like contractility were induced in prometaphase-arrested cells by Cdk1 inhibition. As cells proceeded to “anaphase”, de novo synthesized MTs assembled as central spindle-like structures at the schizont surface (Figure 7A). Even though normal sister chromatid separation did not take place, cells attempted cleavage (Figure S9). RhoA, the main regulator of actin dynamics, was found to accumulate at the cell cortex in a narrow zone in the immediate vicinity of the parasite (Figure 7B). Interestingly, cleavage furrows occurred almost invariably at sites where MT bundles had assembled on the parasite surface (Figure 7C, Figure S9), and the position of host cell chromosomes had little or no influence on this process. These findings indicate that by forming a foundation for Plk1-enrichment and MT polymerization the schizont may help focus MT-associated cytokinesis signals known to stimulate contractile ring assembly and furrow ingression.

**Figure 8:**
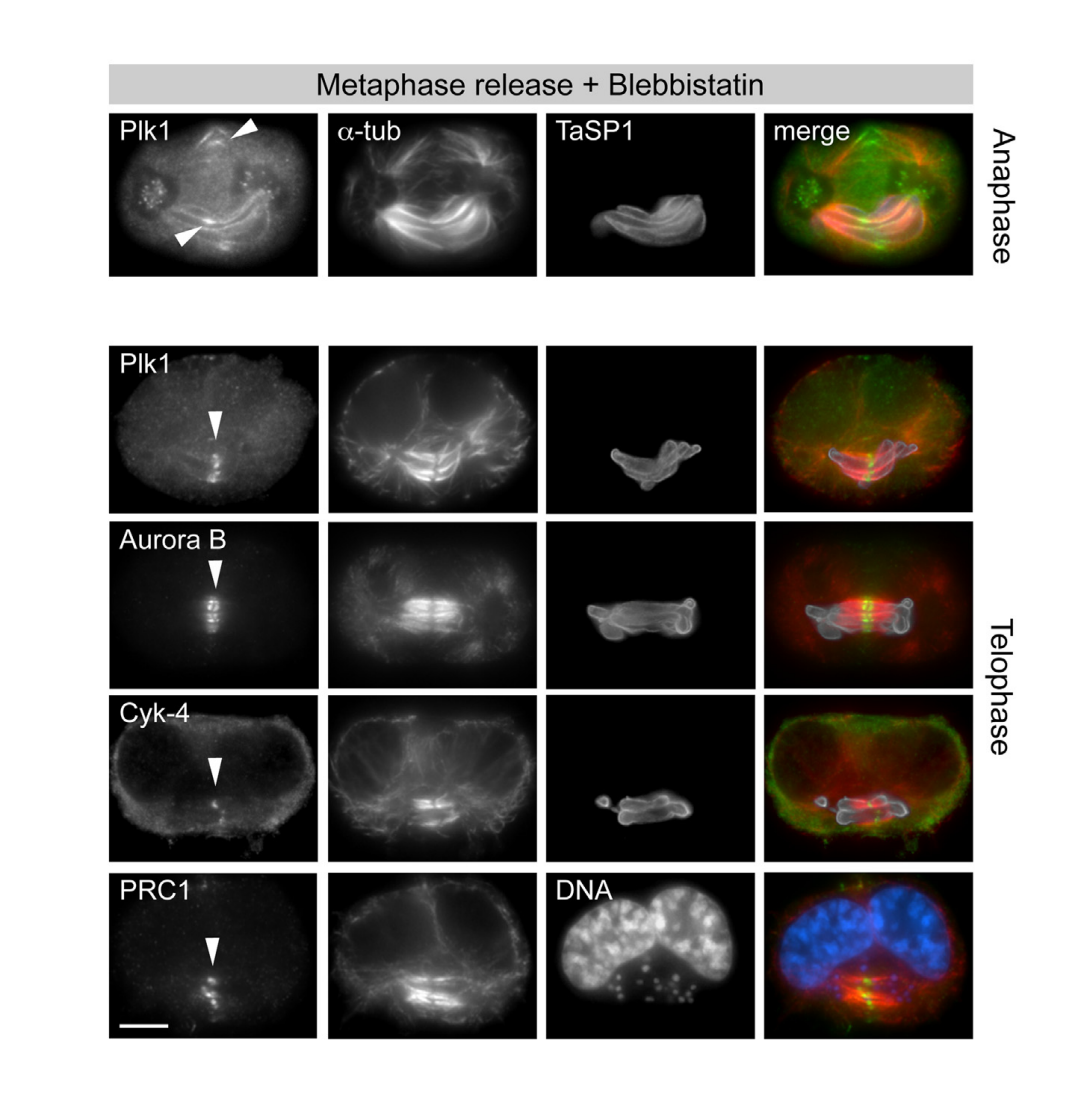
The schizont recruits host cell central spindles to its surface. Cells released for 80 min from MG132-induced metaphase arrest in the presence of the myosin II inhibitor blebbistatin (to prevent furrow ingression) were stained for central spindle proteins (arrowheads) as indicated; microtubules were stained using anti-α-tubulin; DNA was stained with DAPI. Scale bars represent 5 µm.

Prompted by the findings above, we analyzed the central spindle-like structures associated with the parasite in more detail. In bona fide central spindles, anti-tubulin antibodies do not label the zone where antiparallel MT bundles overlap, most likely because of epitope masking caused by the accumulation of central spindle components such as PRC1, centralspindlin, and the chromosomal passenger complex [15]. PRC1 is a major substrate of Plk1 that accumulates in the midzone during anaphase where it participates in central spindle MT bundling. Aurora B, a member of the chromosomal passenger complex, and Plk1 both translocate from kinetochores to the central spindle. There, Plk1 is recruited by PRC1 [20] and Mklp2 [36] and contributes to anaphase spindle elongation and the regulation of cytokinesis [21],[22],[24]–[27]. To avoid potential side effects caused by nocodazole treatment and chemical inhibition of Cdk1, experiments were carried out using cells synchronized in metaphase by MG132, as these possess an intact mitotic spindle and undergo normal anaphase. In addition, to exclude the possibility that colocalization of the central spindle-like structures with the parasite surface merely occurred by apposition induced by furrow contraction, we made use of the myosin II inhibitor blebbistatin, which blocks contraction of the cleavage furrow without affecting mitosis or assembly of the contractile ring [44]. Upon removal of MG132, cells progressed to anaphase and central spindle-like structures assembled on the parasite (Figure 8). The midzone components Plk1, Aurora B, PRC1, and Cyk-4/MgcRacGAP all localized to the centre of parasite-associated central spindle-like structures (arrowheads). Cyk-4/MgcRacGAP is a RhoGAP and member of the centralspindlin complex, which is required for central spindle assembly and also recruits the RhoGEF Ect2 to the central spindle (see review [15] and references therein; [21],[22]). Similar observations were made with cells in which precocious anaphase was induced by Cdk1 inhibition (Figure S10). Together, our findings indicate that the central spindle-like structures assembling at the surface of the schizont resemble bona fide central spindles.

**Figure 9:**
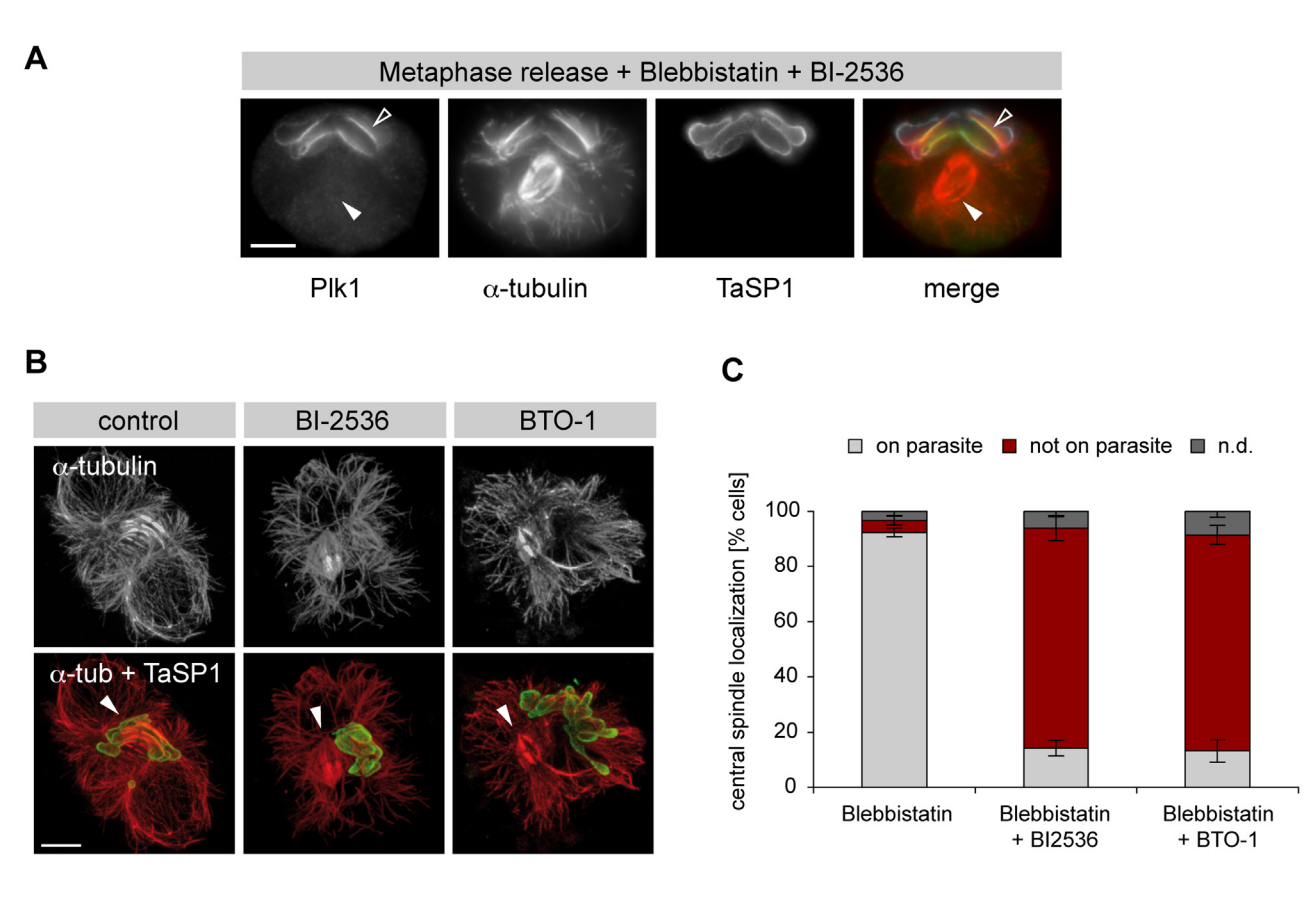
Recruitment of central spindles, but not astral MTs, to the parasite surface requires catalytically active Plk1. The effect of Plk1 inhibition on parasite-central spindle association was monitored by IFM in cells released (80 min) from MG132-induced metaphase arrest. (A) Cells were released from metaphase arrest in the presence of blebbistatin and BI-2536 and stained for Plk1, α-tubulin, and TaSP1. Open arrowheads: parasite-associated Plk1; filled arrowheads: central spindles lacking Plk1. (B) Cells were released from metaphase arrest in the presence of blebbistatin only (control, left panels), blebbistatin and BI-2536 (BI-2536, middle panels), or blebbistatin and BTO-1 (BTO-1, right panels), and stained for α-tubulin and TaSP1. Whole cells are displayed by maximum intensity projection of confocal sections. Arrowheads indicate the position of the central spindle. (C) Cells were released from metaphase arrest in the presence of blebbistatin only, blebbistatin and BI-2536, or blebbistatin and BTO-1. Cells in anaphase and telophase were monitored by IFM for central spindle association with the parasite based on staining of PRC1 and a parasite surface marker. Data represent the mean of three experiments ± SD with *n* = 100 cells/sample; n.d., not determined (includes all cells in anaphase or telophase that could not be classified). Scale bars represent 5 µm.

We next tested whether central spindle association with the parasite surface is dependent on Plk1 catalytic activity. In cells released from metaphase in the presence BI-2536, central spindle formation at the parasite surface was strongly reduced. Instead, central spindles formed in the centre of the cell, independent of the position of the parasite (Figure 9). Plk1 did not localize to the middle section of the central spindles (Figure 9A, filled arrowheads) in BI-2536-treated cells but, as observed before, accumulated on the parasite surface (Figure 9A, open arrowheads). Identical results were obtained using a second, structurally unrelated Plk1 inhibitor, BTO-1, indicating that the effects observed with BI-2536 are not off-target effects (Figure S11A). In Figure 9B, whole cells are displayed by maximum intensity projection of confocal sections. MT staining confirms that, when Plk1 was inhibited by BI-2536 or BTO-1, central spindle assembly was not linked to the location of the parasite in the cytoplasm, and central spindle MT bundles failed to associate with the parasite surface. To facilitate monitoring the effect of Plk1 inhibition on the relative position of parasite and central spindles in more detail, cells were stained with an antibody that recognizes PRC1, an intrinsic marker of central spindles (Figure S11B). Whereas central spindles located at the parasite surface could be observed in >90% of control cells, this was reduced to <20% in cells treated with BI-2536 or BTO-1 (Figure 9C), reflecting a marked decrease in the parasite's affinity for central spindles. Interestingly, BI-2536 did not prevent the interaction of the schizont with mitotic spindle MTs emerging from the spindle poles, and such interactions could still be observed during anaphase. This indicates that Plk1 activity is only required for interaction with the central spindle.

### Interaction with Host Cell Astral and Central Spindles Is Required for Schizont Segregation During Cytokinesis

Although our observations so far are consistent with the hypothesis that schizont interaction with mitotic and central spindle MTs is required for its distribution over the two daughter cells—and thus for parasite maintenance, they do not provide functional evidence to that extent.

During late anaphase, the schizont can still be found associated to host cell MTs emanating from the spindle poles (see for instance Figures 1B, 2A, and 8, 9), and in contrast to schizont interaction with central spindle MTs, this process does not require Plk1 catalytic activity. We tested whether preventing the interaction of the schizont with MTs emanating from the spindle poles or with central spindle MTs interfered with schizont segregation during cytokinesis. Treatment with nocodazole after the onset of anaphase can result in the disassembly of astral MTs, whereas midzone MTs remain differentially stable [45]–[47]. This allowed us to separate the contribution of astral MTs in positioning the parasite from that of central spindle MTs.

**Figure 10:**
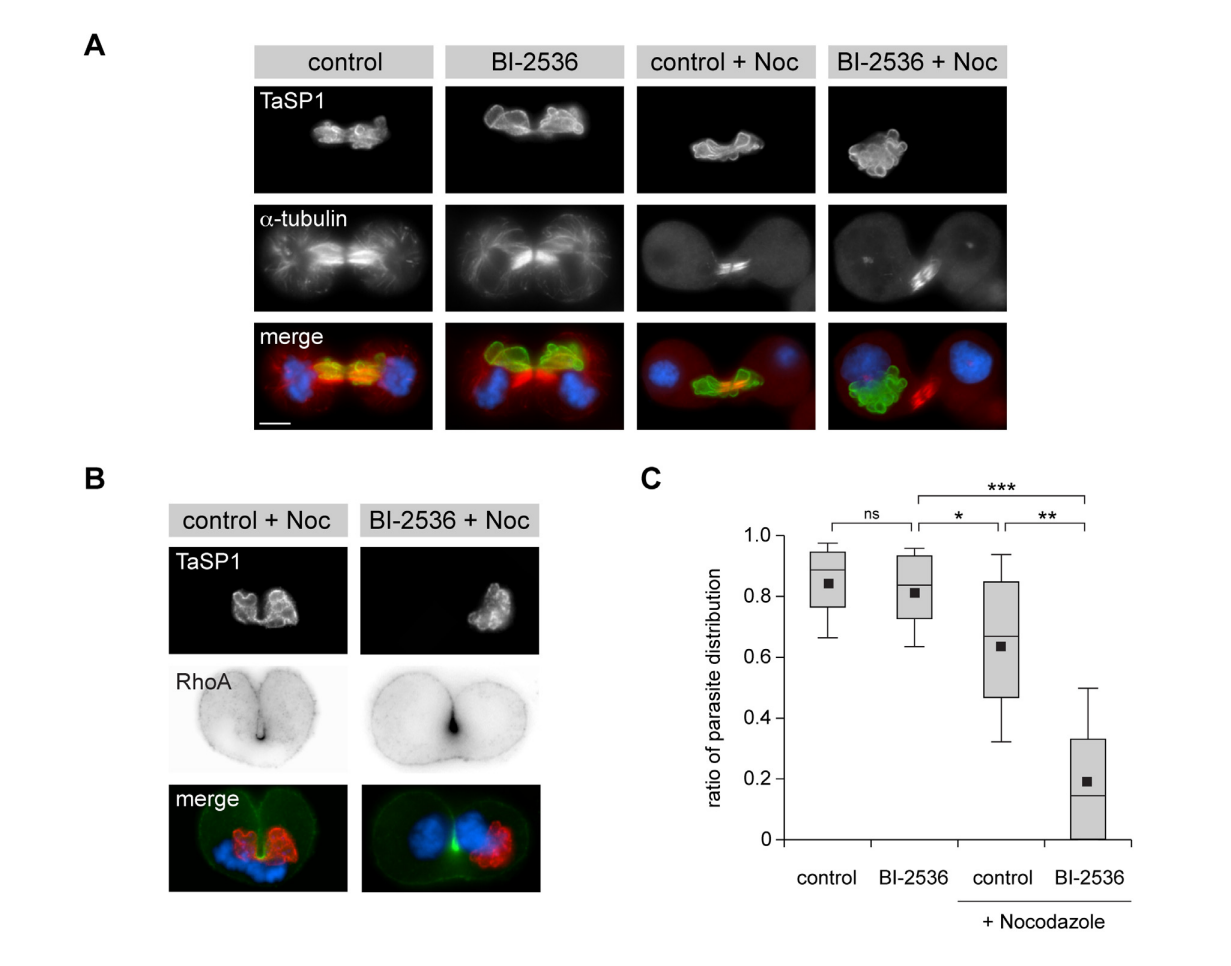
Interaction with host cell astral and central spindles is required for schizont segregation during cytokinesis. The respective role of astral and central spindles for parasite positioning was tested by nocodazole treatment of anaphase host cells and IFM analysis. Cells were released from metaphase arrest in the presence of control solvent DMSO (control) or BI-2536. After 60 min of release, cells were additionally subjected to 10 µM nocodazole over a period of 20 min. (A) Cells were stained for α-tubulin and TaSP1 to determine the position of the parasite relative to the central spindle and cleavage furrow. (B) Micrographs of cells displaying unilateral furrowing. Cells were treated as described in (A) but stained for RhoA and the parasite surface marker TaSP1. DNA was stained with DAPI; scale bars represent 5 µm. (C) Cells were stained as in (A) and microscopic z-stacks of whole cells were generated. Two-dimensional projections were generated to measure the area occupied by the parasite on each side of the cleavage furrow. The ratio of both areas indicates the positioning, with 1.0 representing an equal distribution over the two forming daughter cells. Data were obtained from three experiments with a total of 45 cells per condition. Significance was tested in a *t* test (one-tailed, unpaired, unequal variance); ns, not significant; * *p*<10^−3^; ** *p*<10^−14^; *** *p*<10^−30^.

*T. annulata*-transformed TaC12 cells were released from metaphase arrest for 60 min and then exposed for 20 min to nocodazole. This resulted in the disassembly of astral MTs and the parasite was found in close association with the central spindle (Figure 10A, control + noc). As described [46], in the absence of astral MTs, furrow ingression was often asymmetric as was also reflected by the predominantly unilateral accumulation of RhoA (Figure 10B). Whereas parasite division between the forming daughter cells per se was not affected, the schizont was distributed less evenly than in control cells. This was monitored by measuring the distribution of parasite surface area on either side of the cleavage furrow (Figure 10C).

When added during metaphase, BI-2536 (and other Plk1 inhibitors) potently block cleavage furrow ingression [22],[24],[26]. When Plk1 is inhibited as cells enter anaphase, however, central spindle formation and cleavage furrow ingression can occur, but cells fail to complete cytokinesis [27]. In TaC12 cells, these conditions are met when BI-2536 is added 15 min after release from MG-132-induced metaphase arrest (Figure S12). While BI-2536 treatment prevented the “incorporation” of the parasite into the central spindle, distribution between the forming daughter cells occurred with the same efficiency as observed for control cells (Figure 10A, BI-2636 and Figure 10C). Importantly, however, in cells that did not contain astral spindle MTs, schizont segregation was severely impeded when parasite-central spindle interaction was blocked by BI-2536 treatment (BI-2536+ Noc), and in many cases, the schizont remained completely sequestered on one side of the cleavage furrow. These data strongly indicate that both astral and central spindle MTs help facilitate parasite distribution between the two daughter cells during cytokinesis.

Taken together, these findings underpin the notion that the interaction with host cell astral and central spindle MTs is essential for proper segregation over the two daughter cells.

## Discussion

Coevolving with their hosts, intracellular pathogens have developed a range of ways to use host cell MTs and their associated signaling pathways to their own advantage [48]. This ranges from MT-mediated viral transport in the cell [49],[50] to microtubular network restructuring by parasites like *Toxoplasma*, which reside in a parasitophorous vacuole [51],[52] or by inhabitants of the cytosol like *Trypanosoma cruzi* [53],[54] and *Theileria*. Whereas *T. cruzi* utilizes host cell MTs to facilitate invasion and establishment of the parasite inside the cytosol [53],[54], *Theileria* in addition evolved ways to usurp structures involved in host cell mitosis and cytokinesis. Our findings reveal interesting differences in the strategies used by transforming viruses and *Theileria* to ensure persistence in continuously dividing cells. Thus, while viruses either integrate into the host cell genome or target mitotic chromosomes to guarantee long-term persistence [12], the transforming protozoan *Theileria*, rather than engaging chromosomes, evolved to single out the mitotic apparatus that mediates chromosome segregation and cytokinesis. How the parasite interacts with the host cell MTs or Plk1 during the different stages of the cell cycle is presently not known. TaSE, a protein reported to be secreted by the *T. annulata* schizont, was recently described to co-localize in a punctate manner with host cell MTs [55]. The pattern of schizont/MT interaction we observed, however, clearly differs from that of TaSE. TaSP1 was recently proposed to interact with MTs [56], but the functional relevance is not yet clear.

Considering the multiple levels in space and time at which individual regulator proteins participate in fine-tuning mitosis and cytokinesis, commonly used approaches such as RNA interference or the expression of dominant negative mutants generally do not lend themselves well for studies on the later steps of M phase as they often inhibit important earlier events. This hurdle was eliminated by the recent development of specific small molecule inhibitors [25]–[27],[44],[57] and we exploited the new opportunities, created by these inhibitors, to investigate the interaction of *Theileria* with host mitotic structures during different stages of M phase.

### Cdk1 Regulates Interaction of Plk1 with the Schizont

The biphasic pattern and the way in which Plk1 interacts with the parasite surface are intriguing. Plk1 binding to the parasite can first be observed during host cell G2 as the cell prepares for mitosis. During early mitosis, when Cdk1 becomes fully activated, Plk1 dissociates from the parasite surface to re-accumulate at the onset of anaphase, when Cdk1 is inactivated by the anaphase promoting complex/cyclosome. The inverse correlation between Cdk1 activity and Plk1 interaction with the schizont and the fact that pharmacological inhibition of Cdk1 induces rapid binding of Plk1 to the parasite clearly point toward Cdk1 as a negative regulator. This is reminiscent of Cdk1's known role as a spatio-temporal regulator of Plk1. Depending on the cell cycle stage, location, and kinase substrate, Cdk1 can either create Plk1 binding sites or prevent Plk1 binding (reviewed in [17]). For instance, it has been shown that, in metaphase, Cdk1 prevents Plk1 binding to PRC1 by phosphorylating a site adjacent to the Plk1 docking site [20]; this block is lifted upon Cdk1 inactivation at anaphase. By analogy, it would be possible that Cdk1-mediated phosphorylation of the parasite surface prevents Plk1 from docking. Mammalian Plk1 lacks Cdk1 phosphorylation sites, and a direct modification of Plk1 by Cdk1 can therefore be excluded. It is also conceivable that Plk1 binding to the parasite involves one or more additional proteins, of host or parasite origin, forming a complex that can only bind in the absence of Cdk1 activity.

Transfection experiments revealed that Plk1 binds to the schizont through its PBD. Catalytically inactive Plk1 and the PBD alone both bound to the parasite in cells treated with BI-2536, confirming that Plk1 itself is, in all likelihood, not the priming kinase. This is underpinned by the finding that, in the presence of either BI-2536 or BTO-1, increased—rather than reduced—Plk1 binding to the parasite could be observed. Similar results were obtained using a third, unrelated Plk1 inhibitor. On the other hand, PBD mutants lacking H538/K540, required for electrostatic interactions with the negative charges of phospho-S/T groups, did not bind to the schizont surface, indicative of the involvement of a phosphate group in Plk1 docking. The fact that neither Plk1 nor Cdk1 appear to function as the priming kinase points toward the involvement of another serine/threonine kinase. Although unusual, this is not without precedent; for instance, calmodulin-dependent kinase has been shown to create Plk1 binding sites in meiosis [58]–[60]. Alternatively, it is conceivable that Plk1 binds to a moiety that mimics a Plk1 binding site, which is only accessible when Cdk1 is inactive. Plk1 binding to a substrate without priming phosphorylation has been observed in *Drosophila* for Polo binding to the MT-associated protein Map205; in this case the PBD was found to be required, but not sufficient, for interaction [61]. The nature of the schizont surface protein that provides a docking site for Plk1 is presently not known. Considering the complexity of the interactions between parasite and host cell during mitosis, the participation of several schizont proteins is plausible, and experiments are presently underway to address this topic.

### Parasite-Central Spindle Interactions

While many aspects of central spindle assembly are still enigmatic, a picture is emerging, indicating that central spindles show a high degree of self-organization (see review [15] and references therein). It has been shown that the combined presence of PRC1, centralspindlin, and the chromosomal passenger complex suffices to induce the robust bundling of central spindle MTs. All three complexes required for self-organization can be detected in parasite-associated central spindles. The fact that central spindles can be detected at the parasite surface within a very short time after induction of precocious anaphase (within 10 to 20 min of Cdk1 inactivation) suggests that central spindles either assemble in situ or interact with the schizont immediately after they are formed. In cells in which furrow ingression was blocked by treatment with the myosin II inhibitor blebbistatin, newly formed central spindles were found almost invariably in association with the parasite surface. When Plk1 was inhibited, however, central spindles assembled independently of the position of the schizont. Parasite interaction with astral spindle MTs emanating from the spindle poles, on the other hand, was clearly not affected. Astral spindle MTs have themselves been implicated in central spindle assembly (see reviews [15],[62],[63] and references therein). One plausible explanation could therefore also be that a subpopulation of MTs emanating from the spindle poles form stable interactions with the parasite surface where they subsequently self-organize into central spindles in a Plk1-dependent manner.

Whichever the mechanism, we propose that by interacting with astral MTs emerging from both spindle poles, the schizont is aligned strategically spanning across metaphase chromosomes arranged at the equator of the cell. This interaction in all likelihood also ensures that the parasite remains positioned correctly as the chromosome masses separate during anaphase, at which time a central section of the parasite interacts with midzone MTs that form the central spindle in preparation of the ensuing cytokinesis.

### Cleavage Furrow Formation

MTs have been reported to be the main structural constituent of the spindle apparatus required for induction of cell cleavage [64]. The formation of a contractile ring required for cytokinesis depends on the focused localization of myosin II at the cortex of the cell and coordinated activation of the small GTPase RhoA. Astral MTs contribute by spatially coordinating cortical myosin recruitment generating a region of high contractility at the cell equator [65]–[67], and together with central spindle MTs, they localize RhoA to the cell cortex [68]. The central spindle is thought to provide the platform for RhoA activation. Once recruited to PRC1, Plk1 acts to promote the localization of the RhoGEF, Ect2, to the central spindle by phosphorylating Cyk-4 (MgcRacGAP), which functions as Ect2 anchor and activator. This way, a signaling platform is created that triggers RhoA activation in a narrow zone overlaying the central spindle [68]–[70], regulating the onset of division [21],[22]. In normally dividing *Theileria*-transformed cells, the parasite is almost always found symmetrically partitioned between the separating daughter cells with its middle section “incorporated” into the central spindle and the midbody. Despite its size, the presence of the parasite does not appear to disturb cytokinesis. It is conceivable that by accumulating host cell central spindles at its surface, containing the signaling molecules required for RhoA activation, an uninterrupted interaction between the central spindle and cell cortex is guaranteed allowing flawless furrow ingression at the equator of the cell and unperturbed abscission.

Abscission, the final event in cytokinesis leading to two separate cells, involves vesicle transport and membrane fusion (reviewed in [14]). The centralspindlin complex, found first at the central spindle and subsequently at the midbody, regulates not only acto-myosin ring contraction but also vesicle transport to the cleavage furrow, required for abscission [71]. Our microscopic observations show that, during telophase, a short central section of the parasite is first trapped in the midbody as a narrow tube (see Figure 1C) and is subsequently included in the process of abscission. It is not known whether parasite-own structures provide specific cues in preparation of its abscission or whether such signals derive from the host cell. Whichever, once incorporated into the central spindle/midbody, the parasite does not affect host cell central spindle function or abscission, and from this point onward, schizont cytokinesis appears to be a passive process that is largely controlled by the host cell. This is supported by the fact that, in the absence of host cell abscission, independent parasite division does not take place.

**Figure 11:**
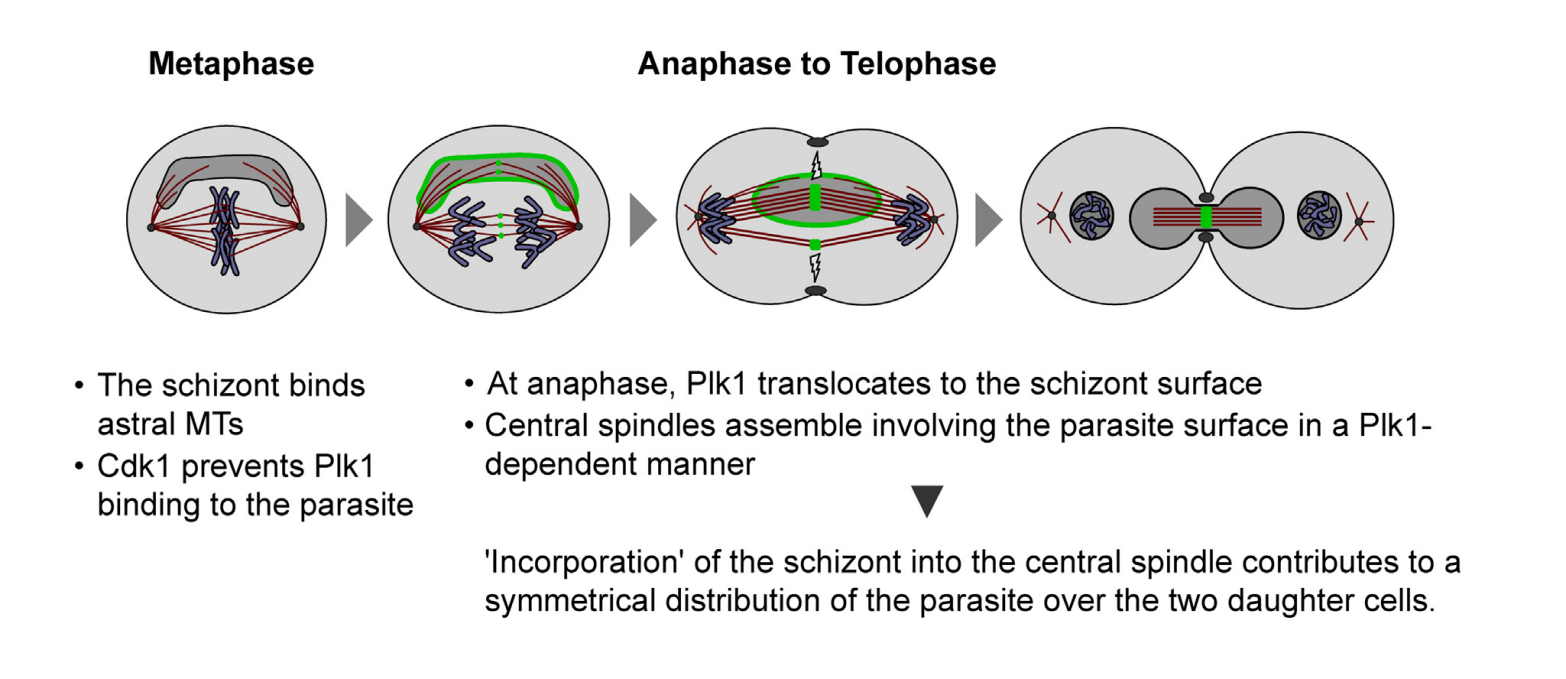
Schematic presentation of parasite interactions with astral and central spindle MTs during host M phase. Green depicts Plk1 binding to the parasite and central spindles.

In summary, we propose a two-step model for the division of *Theileria* schizont between the separating daughter cells, involving first the mitotic spindle and subsequently the central spindle (Figure 11). As the host cell enters mitosis, the schizont binds newly forming MTs that emanate symmetrically from the spindle poles, allowing the schizont to position itself so that it spans the equatorial region of the mitotic cell where host cell chromosomes assemble during metaphase. This step is independent of Plk1 activity as it also takes place in the presence of potent Plk1 inhibitors. During anaphase, the schizont becomes closely associated with central spindles assembling in the midzone between the separating chromosomes. In contrast to the first step, this interaction requires catalytically active Plk1. By “hijacking” the central spindle, an important spatial regulator of cleavage furrow formation, the schizont is strategically positioned to be included in the plane of cell division at each host cell cytokinesis, without disturbing the process.

Thus, while different transforming viruses either integrate into the host cell genome or target mitotic chromosomes to ensure persistence [12], the transforming protozoan *Theileria* evolved to single out the mitotic apparatus that mediates chromosome separation and cytokinesis. Considering the schizont is strictly intracellular [13] and its presence crucial for the constitutive activation of the signaling pathways that drive proliferation and protection against apoptosis (reviewed in [6],[8]), we posit that this process is essential, not only for parasite persistence but also for the exponential expansion of the parasite population.

In a more general context, there is mounting evidence from studies on protein-protein interactions that pathogens have evolved to target host proteins that function as hubs (those involved in many interactions) or bottlenecks (proteins central to many pathways) [72]. In earlier work we provided evidence that *Theileria* hijacks IKK, a central regulator of many NF-κB activation pathways [30]. By scavenging Plk1, a key regulator of mitosis, *Theileria* provides a second striking manifestation of this evolutionary process.

## Materials and Methods

### Cell Culture and Transfections

*Theileria annulata* (TaC12)-infected macrophages were cultured in Leibovitz 15 medium (Gibco) supplemented with 10% foetal calf serum (FCS, Amimed), 10 mM Hepes pH 7.2 (Merck), 2 mM L-glutamine (Gibco), 70 µM β-mercaptoethanol (Merck), and antibiotics (Lonza). The SV40-transformed cell line of *Theileria*-uninfected bovine macrophages (BoMac) was cultured in DMEM Glutamax medium (Gibco) supplemented with 10% FCS and antibiotics.

Plasmids encoding myc-tagged versions of human Plk1 (Figures 5, 6, and S8), including full-length wild-type Plk1, full-length kinase dead Plk1 (K82R), wild-type kinase domain (aa 1–330), wild-type PBD (aa 326–603), and mutant PBD (H538A/K540A), were previously described [43]. Cells were transfected using Lipofectamine 2000 (Invitrogen) following the manufacturer's recommendations. TaC12 cells stably expressing mRFP-α-tubulin (Figure S2) were transfected with the plasmid pmRFP-C1 (a kind gift by Daniel Gerlich, ETH Zürich) and selected using 2 mg/ml G418 (Alexis).

### Synchronizations, Drug Treatments, and Western Blotting

Stocks of the inhibitors BI-2536 (a kind gift by Boehringer Ingelheim, and partly purchased from Axon Medchem), BTO-1, blebbistatin (Sigma) MG132, monastrol, and RO-3306 (Alexis) were prepared in DMSO. DMSO was added at the appropriate concentration to all control samples. Cells were washed in serum-supplemented medium when transferred to another medium. To test the binding of ectopically expressed versions of Plk1 in the presence of Plk1 inhibitor, cells were treated with 100 nM (Figure 6) or 1 µM BI-2536 (Figure S8) immediately after transfection and incubated for 8 h. For the MT re-polymerization assay (Figure 1A), cells were arrested in prometaphase by addition of 0.1 µg/ml nocodazole (Biotrend) for 16 h and harvested by shake-off followed by 30 min treatment with 3 µg/ml nocodazole. These cells were then released in drug-free medium for up to 120 min. For synchronous release from S arrest, cells were treated with 4 mM thymidine (Sigma) for 24 h and transferred into drug-free medium for up to 12 h (Figure S5). To demonstrate the absence of Plk1 from the parasite surface during early mitosis, cells were arrested in prometaphase by 16 h treatment with 0.1 µg/ml nocodazole or 100 µM monastrol and harvested by shake-off (Figure 2B). For the chemical induction of precocious cytokinesis, cells were arrested in prometaphase by 16 h treatment with 0.1 µg/ml nocodazole, harvested by shake-off, washed, and then treated with medium lacking nocodazole but containing 10 µM RO-3306 for up to 60 min (Figures 3A, 7A–C, S9). Alternatively, prometaphase cells were kept in the presence of nocodazole and treated with RO-3306 plus 3 µg/ml nocodazole (Figure 3A, B). To test the requirement of Plk1 catalytic activity for its binding to the parasite surface as well as the effect of Plk1 inhibition on ectopic furrowing (Figures 4B–D, S7F), cells were synchronized in S phase by thymidine block (see above), washed, and immediately treated with 0.1 µg/ml nocodazole or BI-2536 (100 nM or 1 µM) for 15 h. Cells arrested in prometaphase were collected by shake-off and fixed or washed (nocodazole-blocked cells only) and treated with 10 µM RO-3306 or kept in the presence of BI-2536 and additionally subjected to 10 µM RO-3306 for 10–30 min.

To prevent cleavage furrow ingression, RO-3306-treatment was done in the presence of 100 µM blebbistatin (Figure S10). For synchronous release into anaphase, cells were arrested in prometaphase with 0.1 µg/ml nocodazole, harvested by shake-off, transferred into medium containing 20 µM MG132 for 2 h, and finally released from metaphase arrest for 80 min in drug-free medium (Figure 2C) or medium containing 100 µM blebbistatin (Figures 8, 9A–C, S11A, B). For anaphase-specific inhibition of Plk1, these cells were additionally treated with 100 nM or 1 µM BI-2536 or 20 µM BTO-1 during washout (S12A), or 15 min after MG132 washout (Figures 4E, 9A–C, S7D and E, S11A and B, S12A–C). To investigate the role of astral and central spindle MTs for parasite positioning (Figure 10A–C), cells were released from metaphase arrest as described above in the presence of 100 nM BI-2536 (added 15 min after MG132 washout) or inhibitor-free medium. After 60 min release the medium was supplemented with 10 µM nocodazole and cells were incubated for 20 min at 37°C. To test the effect of BI-2536 on spindle maintenance, metaphase-synchronized cells were additionally treated with 100 nM BI-2536 for 160 min (Figure S7B and C).

For the elimination of the parasite from TaC12 cells (Figure S1A and B), cultures were grown 4 d in the presence of 100 ng/ml of the theilericidal drug BW720c [73]. Cells were then subjected to a MT re-polymerization assay as described above. Chemical induction of merogony (Figure S6) was done as previously described [33]. Briefly, cells were cultivated in the presence or absence of 50 µM chloramphenicol (Sigma) for 10 d.

Synchronous entry into M phase (Figure S5) and progression from metaphase to anaphase (Figure 2C) were monitored by immunoblot analysis of protein extracts prepared in RIPA buffer (50 mM Tris-Hcl pH 7.5, 1% NP-40, 0.25% Na-deoxycholate, 150 mM NaCl, 1 mM EDTA, 1 mM PMSF, 1× Roche Complete protease inhibitor cocktail, 1 mM NaF, 1 mM Na_3_VO_4_). Primary antibodies were mouse monoclonal anti-cyclin B1 (clone GNS-11, Pharmingen), anti-Plk1 (clone 35–206, Calbiochem), anti-securin (clone DCS-280, MBL), anti-α-tubulin (clone DM1A, Sigma), as well as rabbit polyclonal anti-phospho-Ser10 histone H3 (Upstate) and anti-histone deacetylase 1 (Santa Cruz).

### Immunofluorescence Microscopy and Time-Lapse Imaging

Interphase cells were grown on coverslips, and cells harvested by mitotic shake-off were seeded on poly-L-lysine coated coverslips (Sigma). Samples were fixed with 4% paraformaldehyde in PBS for 10 min at room temperature (for Aurora B, c-myc, Plk1, TamR1, TaSP, and α-tubulin staining), with methanol for 10 min at −20°C (for Cyk-4 and PRC1 staining) or with 10% trichloroacetic acid on ice for 15 min (for RhoA staining). Cells were subsequently permeabilized in 0.2% Triton X-100 (prepared in PBS) for 10 min at room temperature. Antibody incubations were done in PBS containing 10% heat-inactivated FCS, DNA was stained with DAPI (Molecular Probes), and cells were mounted using Glycergel (Dako). Widefield microscopy was done on a Nikon Eclipse 80i microscope equipped with a Retiga 2000R CCD camera (Qimaging) using 60× and 100× Plan Apo objectives (Nikon) and Openlab 5 software (Improvision). For confocal microscopy, a Leica TCS SP2 system was used, equipped with an acousto-optical beam splitter, a 63× Plan Apo objective (Leica), and Leica confocal software. Images were processed using Photoshop (Adobe) or Imaris (Bitplane) software. To measure the distribution of the parasite in dividing cells after nocodazole wash-in (Figure 10A–C), whole cells were stained for TaSP1/α-tubulin and recorded using z-stacks. Two-dimensional projections were generated and the parasite area on each side of the cleavage furrow was measured using Openlab software.

The following antibodies were used: mouse monoclonal anti-Aurora B (AIM-1, clone 6, BD Transduction Laboratories), anti-c-myc (clone 9E-10, Santa Cruz), anti-Plk1 (clone 35–206, Calbiochem), anti-Rho A (clone 26C4, Santa Cruz), anti-α-tubulin (clone DM1A, Sigma) and 1C12, which detects the *T. annulata* schizont surface (kindly provided by Brian Shiels, University of Glasgow), as well as rabbit polyclonal anti-PRC1 (kindly provided by Francis Barr, University of Liverpool), anti-TamR1 (Brian Shiels, University of Glasgow) [74], and the schizont surface marker anti-TaSP (kindly provided by Jabbar Ahmed, Borstel Research Center) [28], goat polyclonal anti-Cyk-4 (MgcRacGAP, Abcam), and rat monoclonal anti-α-tubulin (Abcam). Appropriate (isotype-specific) secondary antibodies conjugated with either Marina Blue, Alexa-Fluor 488, Alexa-Fluor 594, or Texas Red (Molecular Probes) were used.

To generate kymographs of TaC12 cells stably expressing mRFP-α-tubulin (Figure S2), cells were synchronized in prometaphase using monastrol (see above) and kept in the presence of the drug during time-lapse imaging. Fluorescence was recorded at 30 s intervals over a period of 20 min and kymographs were generated from these data with a width of 5 pixels using NIS Elements imaging software (Nikon). Immediately after imaging, cells were fixed and stained (see above) to identify the position of the parasite in the previously recorded cells. To determine the percentage of cells displaying furrow ingression after release into anaphase in the presence of 100 nM BI-2536 (S12A), cells were synchronized and drug-treated as described above and observed by time-lapse imaging in 10 min intervals over a period of 4 h. Time-lapse imaging was done using a TE2000E-PFS microscope (Nikon) equipped with a Plan Fluor 20×, 60× objective (Nikon), Orca ER CCD camera (Hamamatsu), and incubation chamber (Life Imaging Services).

### Flow Cytometry

To test the effect of BI-2536 on cell cycle progression of TaC12 cells (Figure S7A), unsynchronized cultures were cultured in the presence of 100 nM BI-2536 or the equivalent volume of DMSO for 20 h. To monitor the effect of Plk1 inhibition on progression of M phase in TaC12 cells (Figure 4A), cultures were synchronized in S phase as described above and released for 20 h in the presence of 100 nM or 1 µM BI-2536. Cell suspensions were fixed in 80% ethanol at −20°C o/n followed by treatment with 200 µg/ml RNaseA in PBS at 37°C for 30 min. Finally, cells were stained in DAPI staining solution (100 mM Tris-HCl, pH 7.5, 150 mM NaCl, 1 mM CaCl_2_, 0.5 mM MgCl_2_, 0.1% NP-40, 3 µM DAPI), and cellular DNA content was measured using a BD LSR II and BD FACS Diva software (Becton-Dickinson).

## Supporting Information

Figure S1 **Microtubule repolymerization and spindle midzones in unparasitized macrophages.** (A) Cells from which the parasite was eliminated by treatment with the theilericidal compound BW720c were subjected to a microtubule repolymerization assay. Samples were fixed and stained for α-tubulin and the parasite surface protein TaSP1. The absence of TaSP1 staining confirms that the parasite had been eliminated. (B) Central spindles in BW720c-treated cells stained as in (A). (C) Central spindle and midbody formation in transformed bovine macrophages (BoMac) stained with anti-PRC1 and anti-α-tubulin. DNA was stained with DAPI; scale bars represent 5 µm.
(1.78 MB TIF)

Figure S2 **Mitotic microtubules are stably associated with the parasite surface.** (A) IFM micrograph of the monastrol-treated cell observed by time-lapse imaging shown in (B). The cell was fixed and stained with anti-α-tubulin and anti-TaSP1 immediately after live imaging. Scale bar represents 5 µm. (B) *T. annulata*-transformed TaC12 cells stably expressing mRFP-α-tubulin were synchronized in prometaphase by monastrol treatment and observed by time-lapse imaging in the presence of the drug. The two left panels show the same frame of an image sequence recorded in 30 s intervals over 20 min. Data from 40 frames were used to generate kymographs. White rectangles show the regions chosen for the kymographs; p indicates the source used for the kymograph of microtubules associated with the parasite surface and c that for free microtubules. Results for c and p are shown in the righthand panels. Data are representative for 12 cells observed under identical conditions. Vertical bars represent 2 µm; horizontal bars represent 10 min.
(0.81 MB TIF)

Figure S3 **Plk1 association with the surface of the** ***T. annulata*** **schizont during different stages of the cell cycle.** Plk1 recruitment to the parasite surface was analyzed by immunofluorescence microscopy using anti-Plk1 and anti-TaSP1; DNA was stained with DAPI. Closed arrowheads point at Plk1 binding to the schizont. Plk1 can also be detected on host cell structures (open arrows) including centromeres/kinetochores (Prometaph), spindle poles (Metaphase), central spindles (Ana-/Teloph.), and midbody (Teloph./G1). Scale bar represents 5 µm.
(2.28 MB TIF)

Figure S4 **Schematic representation of synchronization experiments (A) and mammalian stages of the *Theileria* life cycle (B).** S, synthesis phase; G2, gap 2 phase; P, prophase; PM, prometaphase; M, metaphase; A, anaphase; T, telophase; C, cytokinesis; Cdk1, cyclin-dependent kinase 1.
(1.47 MB TIF)

Figure S5 **Monitoring Plk1 binding to the schizont in cells released from S phase arrest.** *T. annulata*-transformed cells were synchronized in early S phase by thymidine block. At the indicated times after release, cells were examined by IFM for Plk1 binding to the parasite surface and lysates were prepared for immunoblot analysis. Data are presented as the percentage of cells containing parasites with surface-bound Plk1 in different cell cycle stages as indicated (*n* = 200 cells/sample). Immunoblot: anti-Plk1 was used to follow the increase in Plk1 expression; anti-phospho-Histone H3 (P-H3) was used to monitor entry into M phase; α-tubulin (α-tub) was monitored as a loading control for both supernatant and pellet of each lysate. Samples of time points 0–7 h and 8–12 h were run on separate gels.
(0.38 MB TIF)

Figure S6 **Plk1 binding to the parasite surface is downregulated during merogony.** Top panels: *T. annulata*-transformed TaC12 cell in late G2 phase harboring a schizont with Plk1 bound to its surface. The transforming schizont does not express TamR1, a marker for merogony; parasite and host cell nuclei were stained with DAPI. Middle panels: TaC12 cell containing schizonts in two stages of differentiation: partial merogony (closed arrow) and advanced merogony (open arrow); cells were stained for expression of TamR1 and Plk1. Squared areas are shown at higher magnification. Bottom panels: TaC12 cell containing a parasite in an advanced stage of merogony. Scale bar represents 5 µm.
(2.12 MB TIF)

Figure S7 **Effects of BI-2536 treatment on M phase progression in** ***T. annulata*** **-transformed cells.** (A) Unsynchronized TaC12 cells (upper panel) or TaC12 cells cultured for 20 h in the presence of 100 nM BI-2536 (lower panel) were analyzed by flow cytometry. 2N, G1 phase; 4N, G2/M phase. (B) Metaphase-synchronized TaC12 cells were either kept in the presence of proteasomal inhibitor (MG132) or additionally treated with 100 nM BI-2536 (MG132 + BI-2536). Cells were harvested after 160 min and bi-/monopolar spindles were quantified by IFM; n.d. indicates cells that could not be classified; data represent 250 cells/sample. (C) Micrographs show representative *T. annulata*-infected cells with bipolar metaphase plate (MG132) or collapsed monopolar spindle (MG132 + BI-2536) that were quantified in (B). Cells were stained for Plk1 and α-tubulin and analyzed by IFM. DNA was stained with DAPI; arrowhead indicates the position of the parasite; scale bar represents 5 µm. (D) Metaphase-synchronized TaC12 cells synchronously released into anaphase and treated with DMSO (control) or 100 nM BI-2536 (BI-2536) at 15 min of release. Cells were analyzed after 4 h by IFM and the abundance of mononucleate and binucleate cells was determined for both samples; data represent 250 cells/sample. (E) Micrographs showing cells quantified in (D). Scale bar represents 10 µm. (F) S phase-synchronized TaC12 cells were released in the presence of nocodazole or 100 nM BI-2536 for 15 h and cells arrested in prometaphase were harvested from both cultures. Nocodazole-blocked prometaphase cells were either kept in the presence of the drug (Noc) or washed and treated with Cdk1 inhibitor for 30 min (RO-3306). Prometaphase cells obtained upon BI-2536 treatment were kept in the presence of 100 nM BI-2536 and additionally treated with Cdk1 inhibitor for 30 min (RO + BI). Cells were stained for RhoA and the occurrence of ectopic furrow ingression quantified (100 cells/sample) by IFM; DNA was stained with DAPI; scale bar represents 5 µm.
(2.11 MB TIF)

Figure S8 **Ectopically expressed Plk1 PBD and catalytically inactive Plk1 can associate with the parasite surface in the presence of high doses of the Plk1 inhibitor BI-2536.** *T. annulata*-infected cells were transfected with plasmids encoding myc-tagged versions of Polo-box domain (myc-PBD, wild type), H538A/K540A mutant PBD (myc-PBD H538A/K540A), or catalytically inactive Plk1 (myc-Plk1 K82R) and cultured in the presence or absence of BI-2536 at a concentration of 1 µM. Cells were analyzed by IFM using anti-myc and anti-TaSP1 antibodies. Scale bar represents 5 µm.
(0.86 MB TIF)

Figure S9 **Cleavage furrow ingression occurs at sites where parasite-associated central spindle MTs assemble.** *T. annulata*-transformed cells were synchronized in prometaphase, washed, and precocious anaphase and premature cytokinesis were induced by immediately blocking Cdk1 activity using the specific inhibitor RO-3306 (30 min). The formation of central spindles was monitored using anti-α-tubulin and anti-Plk1. The parasite was visualized using anti-TaSP1. Scale bar represents 20 µm.
(1.07 MB TIF)

Figure S10 **The** ***Theileria*** **schizont recruits central spindles to its surface.** *T. annulata*-transformed cells were synchronized in prometaphase and precocious anaphase induced by blocking Cdk1 using the inhibitor RO-3306. Cleavage furrow contraction was prevented by treatment with the myosin II inhibitor blebbistatin. Parasite-associated central spindles were analyzed for the presence of central spindle-specific proteins such as Plk1, Aurora B, Cyk-4, and PRC1 as indicated. MTs were visualized using anti-α-tubulin, and the schizont was stained using anti-TaSP1; in the lower panels, DNA was stained with DAPI. Arrowhead indicates the position of the parasite. Scale bar represents 5 µm.
(2.34 MB TIF)

Figure S11 **Recruitment of central spindle, but not astral, MTs to the parasite surface requires catalytically active Plk1.** (A) *T. annulata*-transformed TaC12 cells were released for 80 min from metaphase arrest in the presence of the Plk1 inhibitor BTO-1 or control solvent. Cleavage furrow contraction was blocked by treatment with the myosin II inhibitor blebbistatin. Anti-α-tubulin was used to identify central spindles and parasite-associated MTs emanating from the spindle poles. Open arrowheads point at Plk1 binding to the schizont surface; closed arrowheads indicate central spindles lacking Plk1 in the central section. (B) Representative micrographs of IFM stainings that were used to quantitate the association of the parasite with central spindles in control cells and cells treated with Plk1 inhibitors (histogram Figure 9C). Cells were stained for the central spindle marker PRC1 and 1C12, a mouse monoclonal antibody that detects the surface of *T. annulata* schizonts; DNA was stained with DAPI. In cells treated with Plk1 inhibitor, the central spindle, detected by its marker PRC1, is not associated with the parasite. Scale bars represent 5 µm.
(1.65 MB TIF)

Figure S12 **Effects of BI-2536 on furrow ingression in TaC12 cells.** (A) *T. annulata*-infected cells were synchronized in metaphase using the proteasomal inhibitor MG132 and synchronously released into anaphase. Cells were treated with DMSO (control) or 100 nM BI-2536 (0 min) during MG132 washout; alternatively, cells were first exposed to 100 nM BI-2536, 15 min after MG132 washout (15 min). The histogram shows the abundance of furrow ingression in cells monitored by time-lapse imaging over a period of 4 h after MG132 washout. Additionally, cells were harvested at 80 min after MG132 washout, fixed, and analyzed by IFM (lower panel). Data are presented as the percentage of cells showing cleavage furrow ingression (ingressed), cells that lacked any furrow ingression (binucleation), or cells showing collapsed monoplar spindles (monopolar); n.d. denotes cells that could not be classified; data represent 200 cells/sample (time-lapse) or 300 cells/sample (fixed). (B) Micrographs of cells in anaphase or telophae obtained in (A) that were released in the presence of DMSO (control) or 100 nM BI-2536. Cells were stained for Plk1 as well as α-tubulin and analyzed by IFM; DNA was stained with DAPI. (C) Micrographs of representative cells obtained in (A). Cells were stained for RhoA and analyzed by IFM; DNA was stained with DAPI. Scale bars represent 5 µm.
(1.87 MB TIF)

## Abbreviations

Cdk: cyclin-dependent kinase
DAPI: 4′,6-diamidino-2-phenylindole
DMSO: dimethyl sulfoxide
HDAC1: histone deacetylase 1
KDom: kinase domain
MT: microtubule
Noc: nocodazole
PBD: polo-box domain
P-H3: phospho-S10 histone H3
Plk: Polo-like kinase
PRC1: protein regulator of cytokinesis 1
TamR1: *Theileria annulata* rhoptry protein 1
TaSP1: *Theileria annulata* surface protein 1
wt: wild type
