# Supplementary figures and images for: The Transforming Parasite Theileria Co-opts Host Cell Mitotic and Central Spindles to Persist in Continuously Dividing Cells
Source: PLoS Biol. 2010 Sep 28;8(9):e1000499. doi: 10.1371/journal.pbio.1000499 (PMC2946958; doi:10.1371/journal.pbio.1000499)

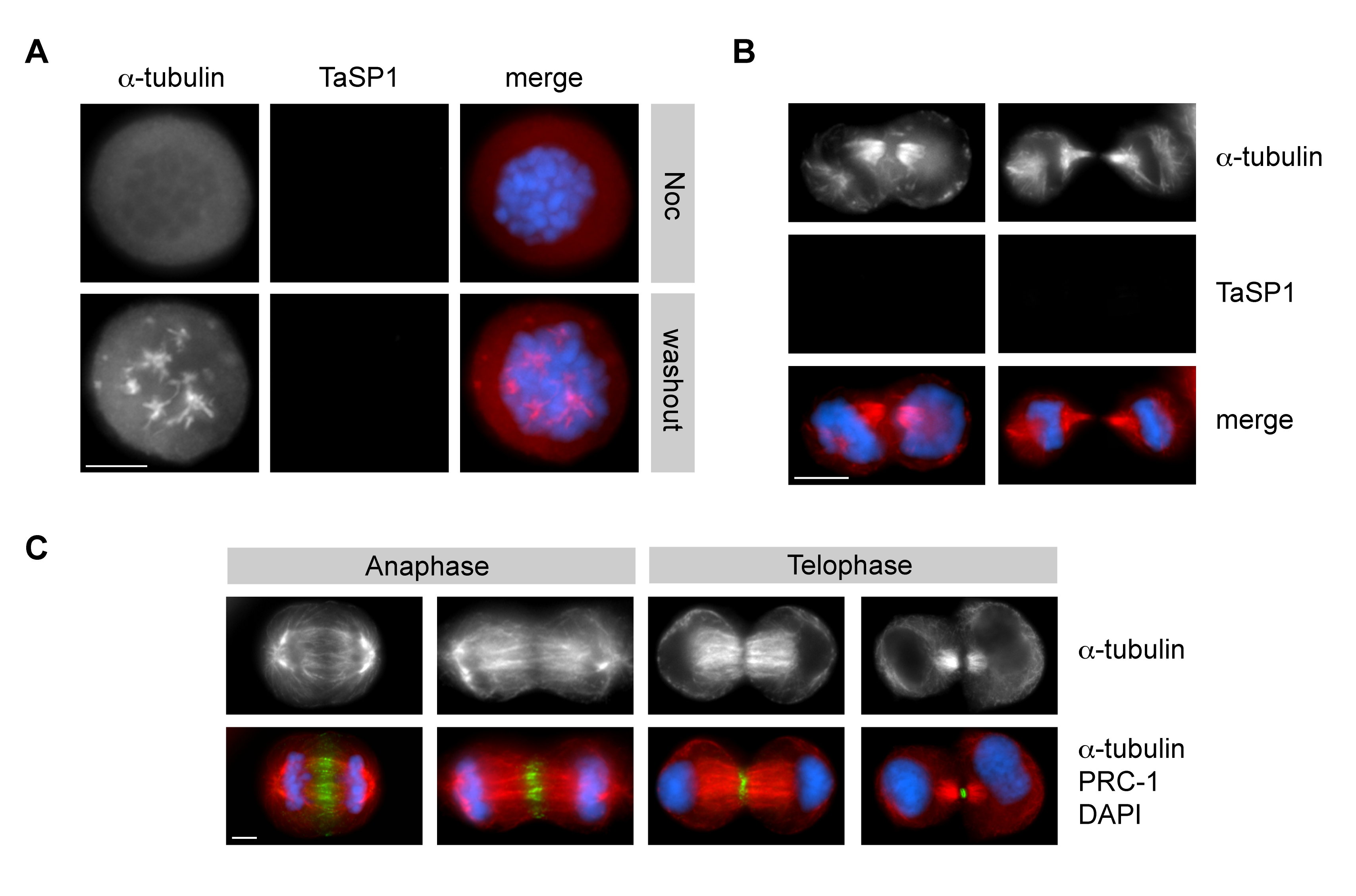

Supplement: Figure S1 — Microtubule repolymerization and spindle midzones in unparasitized macrophages. (A) Cells from which the parasite was eliminated by treatment with the theilericidal compound BW720c were subjected to a microtubule repolymerization assay. Samples were fixed and stained for α-tubulin and the parasite surface protein TaSP1. The absence of TaSP1 staining confirms that the parasite had been eliminated. (B) Central spindles in BW720c-treated cells stained as in (A). (C) Central spindle and midbody formation in transformed bovine macrophages (BoMac) stained with anti-PRC1 and anti-α-tubulin. DNA was stained with DAPI; scale bars represent 5 µm. (1.78 MB TIF) [file pbio.1000499.s001.tif]

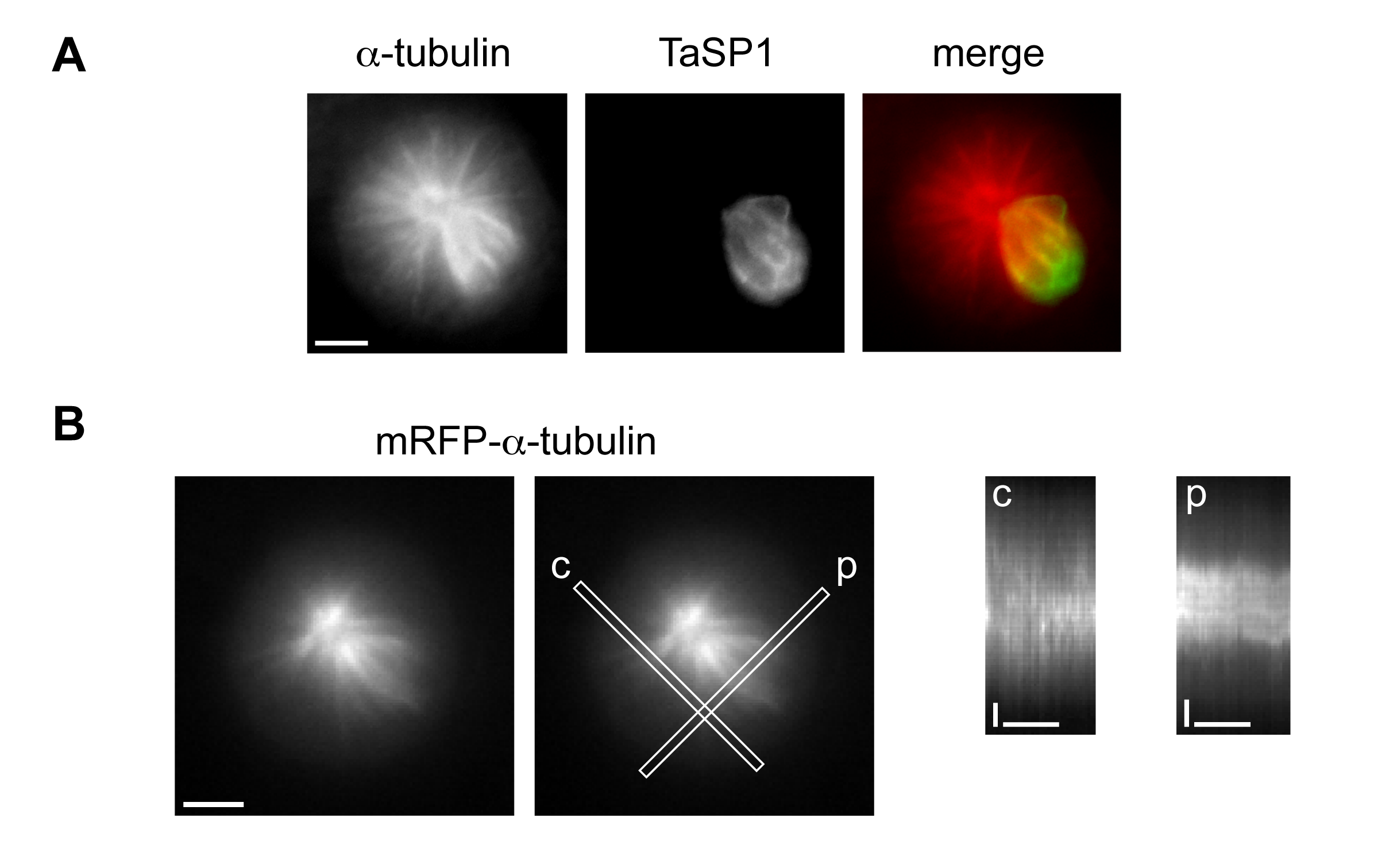

Supplement: Figure S2 — Mitotic microtubules are stably associated with the parasite surface. (A) IFM micrograph of the monastrol-treated cell observed by time-lapse imaging shown in (B). The cell was fixed and stained with anti-α-tubulin and anti-TaSP1 immediately after live imaging. Scale bar represents 5 µm. (B) T. annulata-transformed TaC12 cells stably expressing mRFP-α-tubulin were synchronized in prometaphase by monastrol treatment and observed by time-lapse imaging in the presence of the drug. The two left panels show the same frame of an image sequence recorded in 30 s intervals over 20 min. Data from 40 frames were used to generate kymographs. White rectangles show the regions chosen for the kymographs; p indicates the source used for the kymograph of microtubules associated with the parasite surface and c that for free microtubules. Results for c and p are shown in the righthand panels. Data are representative for 12 cells observed under identical conditions. Vertical bars represent 2 µm; horizontal bars represent 10 min. (0.81 MB TIF) [file pbio.1000499.s002.tif]

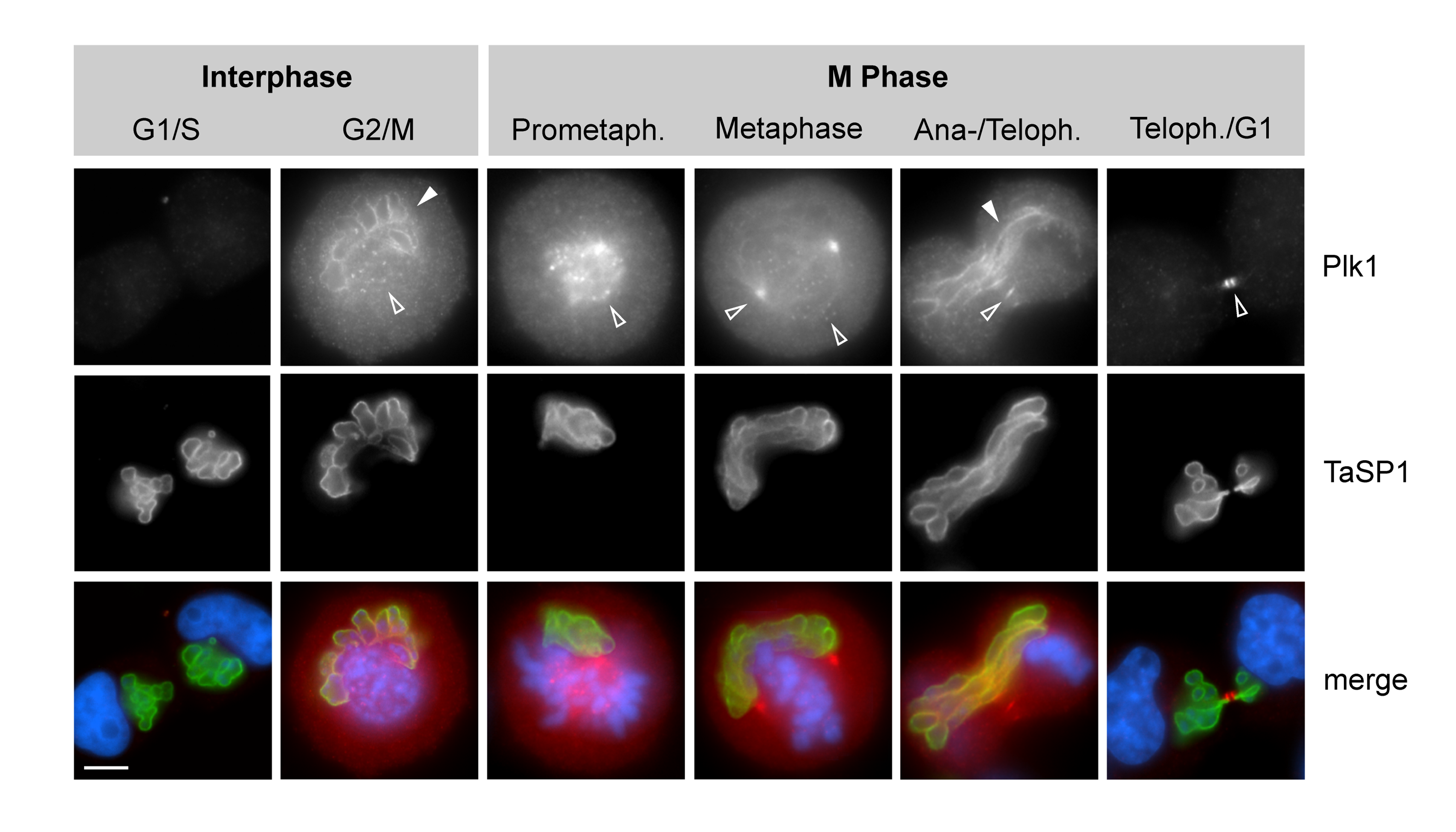

Supplement: Figure S3 — Plk1 association with the surface of the T. annulata schizont during different stages of the cell cycle. Plk1 recruitment to the parasite surface was analyzed by immunofluorescence microscopy using anti-Plk1 and anti-TaSP1; DNA was stained with DAPI. Closed arrowheads point at Plk1 binding to the schizont. Plk1 can also be detected on host cell structures (open arrows) including centromeres/kinetochores (Prometaph), spindle poles (Metaphase), central spindles (Ana-/Teloph.), and midbody (Teloph./G1). Scale bar represents 5 µm. (2.28 MB TIF) [file pbio.1000499.s003.tif]

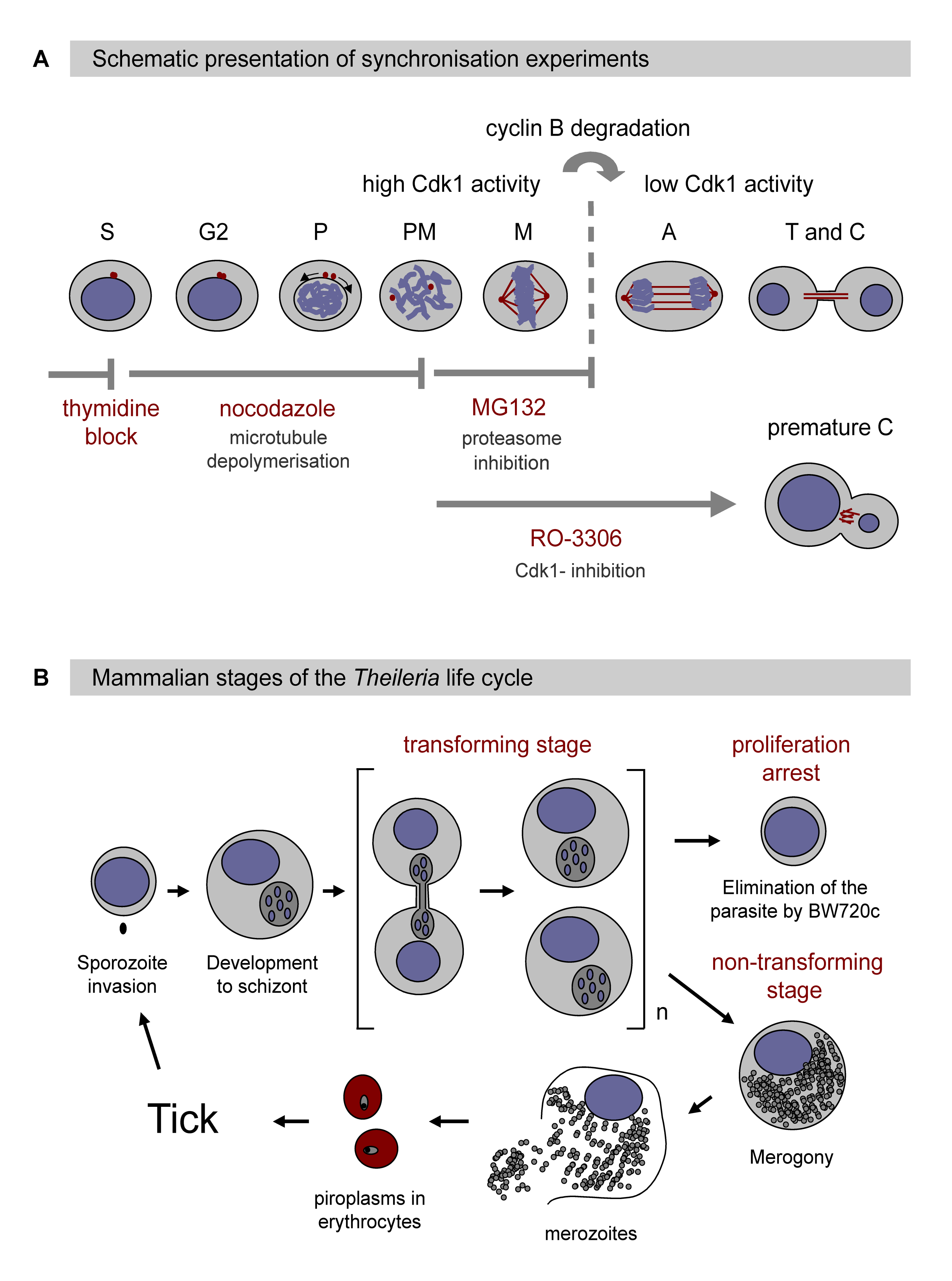

Supplement: Figure S4 — Schematic representation of synchronization experiments (A) and mammalian stages of the Theileria life cycle (B). S, synthesis phase; G2, gap 2 phase; P, prophase; PM, prometaphase; M, metaphase; A, anaphase; T, telophase; C, cytokinesis; Cdk1, cyclin-dependent kinase 1. (1.47 MB TIF) [file pbio.1000499.s004.tif]

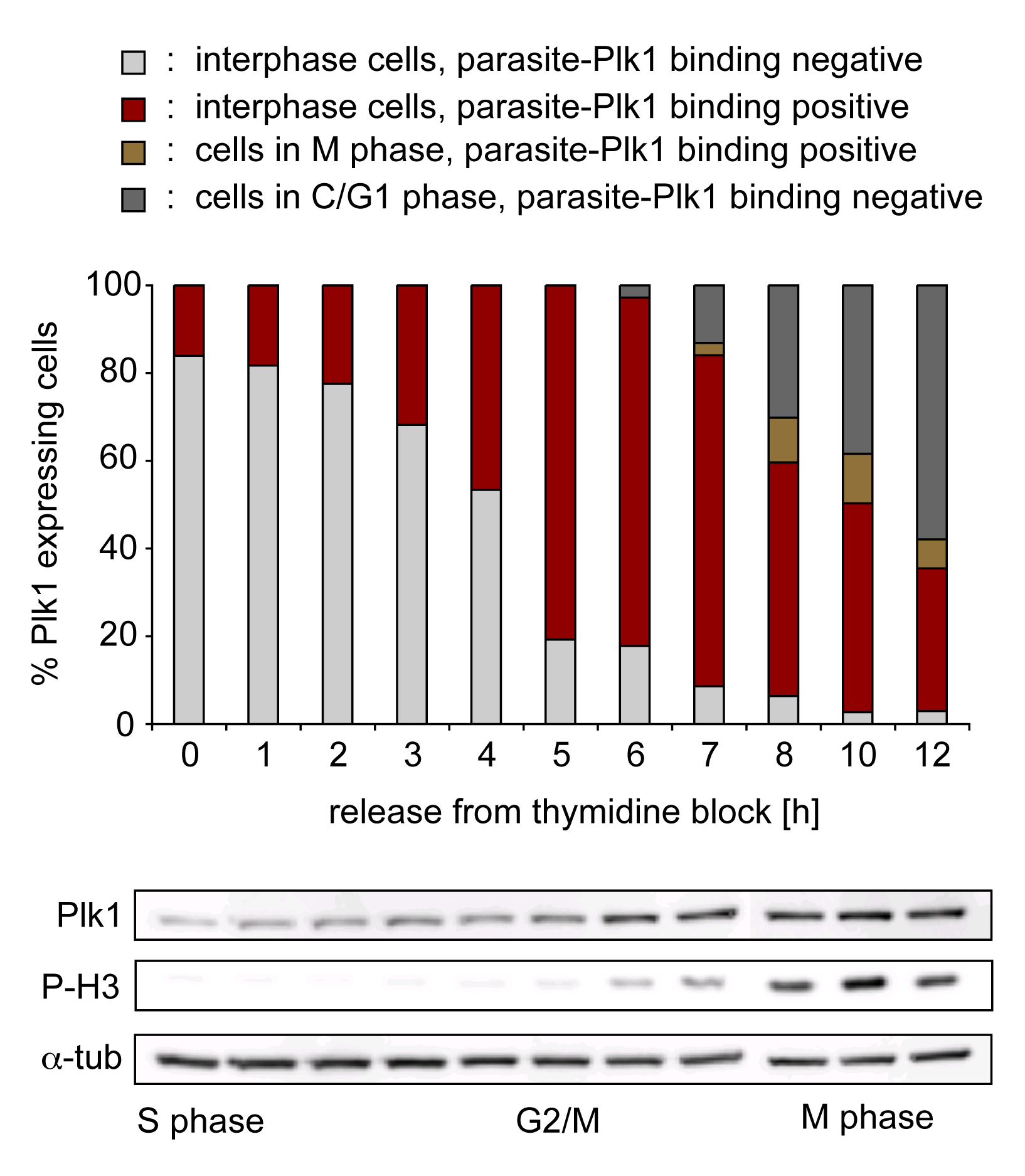

Supplement: Figure S5 — Monitoring Plk1 binding to the schizont in cells released from S phase arrest. T. annulata-transformed cells were synchronized in early S phase by thymidine block. At the indicated times after release, cells were examined by IFM for Plk1 binding to the parasite surface and lysates were prepared for immunoblot analysis. Data are presented as the percentage of cells containing parasites with surface-bound Plk1 in different cell cycle stages as indicated (n = 200 cells/sample). Immunoblot: anti-Plk1 was used to follow the increase in Plk1 expression; anti-phospho-Histone H3 (P-H3) was used to monitor entry into M phase; α-tubulin (α-tub) was monitored as a loading control for both supernatant and pellet of each lysate. Samples of time points 0–7 h and 8–12 h were run on separate gels. (0.38 MB TIF) [file pbio.1000499.s005.tif]

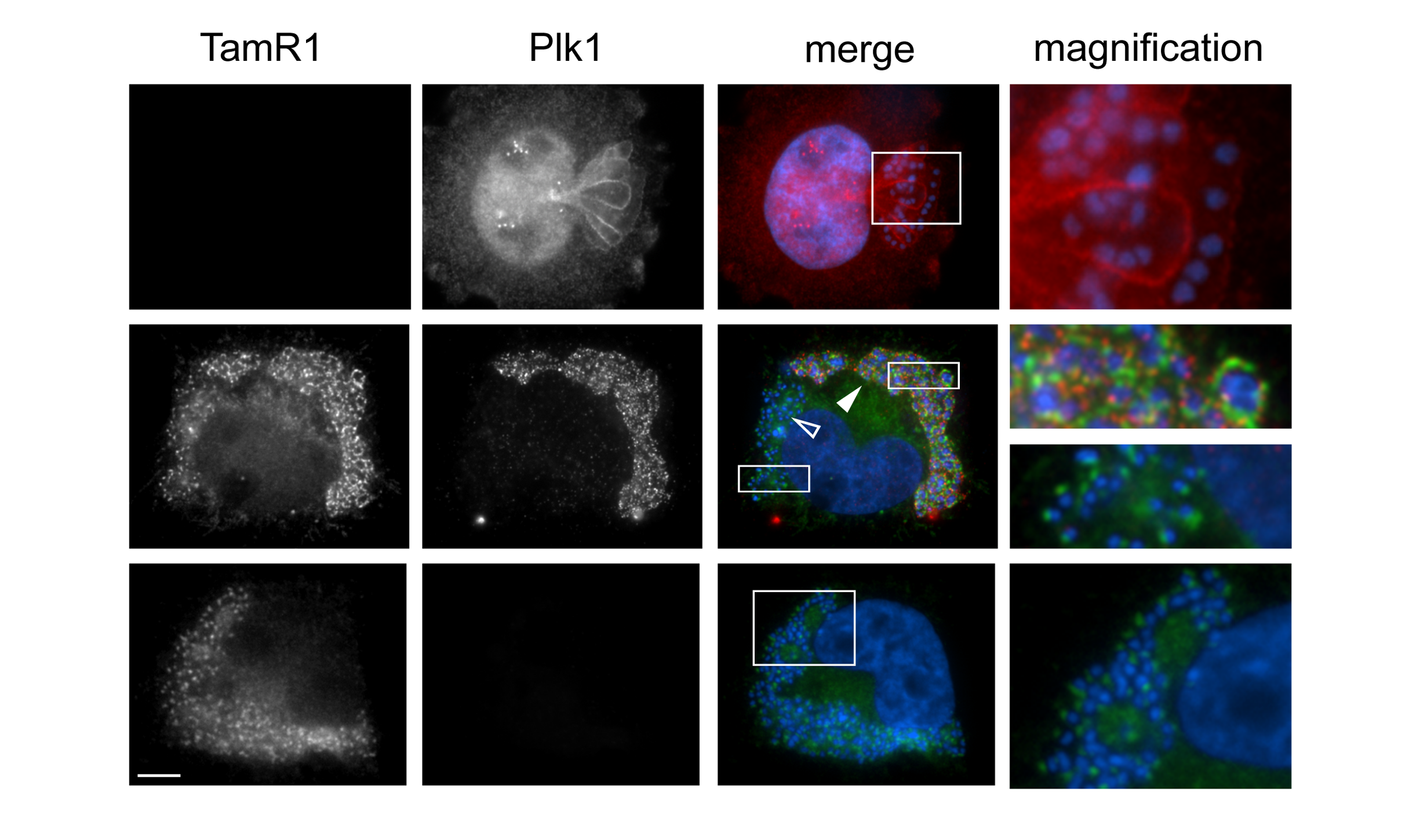

Supplement: Figure S6 — Plk1 binding to the parasite surface is downregulated during merogony. Top panels: T. annulata-transformed TaC12 cell in late G2 phase harboring a schizont with Plk1 bound to its surface. The transforming schizont does not express TamR1, a marker for merogony; parasite and host cell nuclei were stained with DAPI. Middle panels: TaC12 cell containing schizonts in two stages of differentiation: partial merogony (closed arrow) and advanced merogony (open arrow); cells were stained for expression of TamR1 and Plk1. Squared areas are shown at higher magnification. Bottom panels: TaC12 cell containing a parasite in an advanced stage of merogony. Scale bar represents 5 µm. (2.12 MB TIF) [file pbio.1000499.s006.tif]

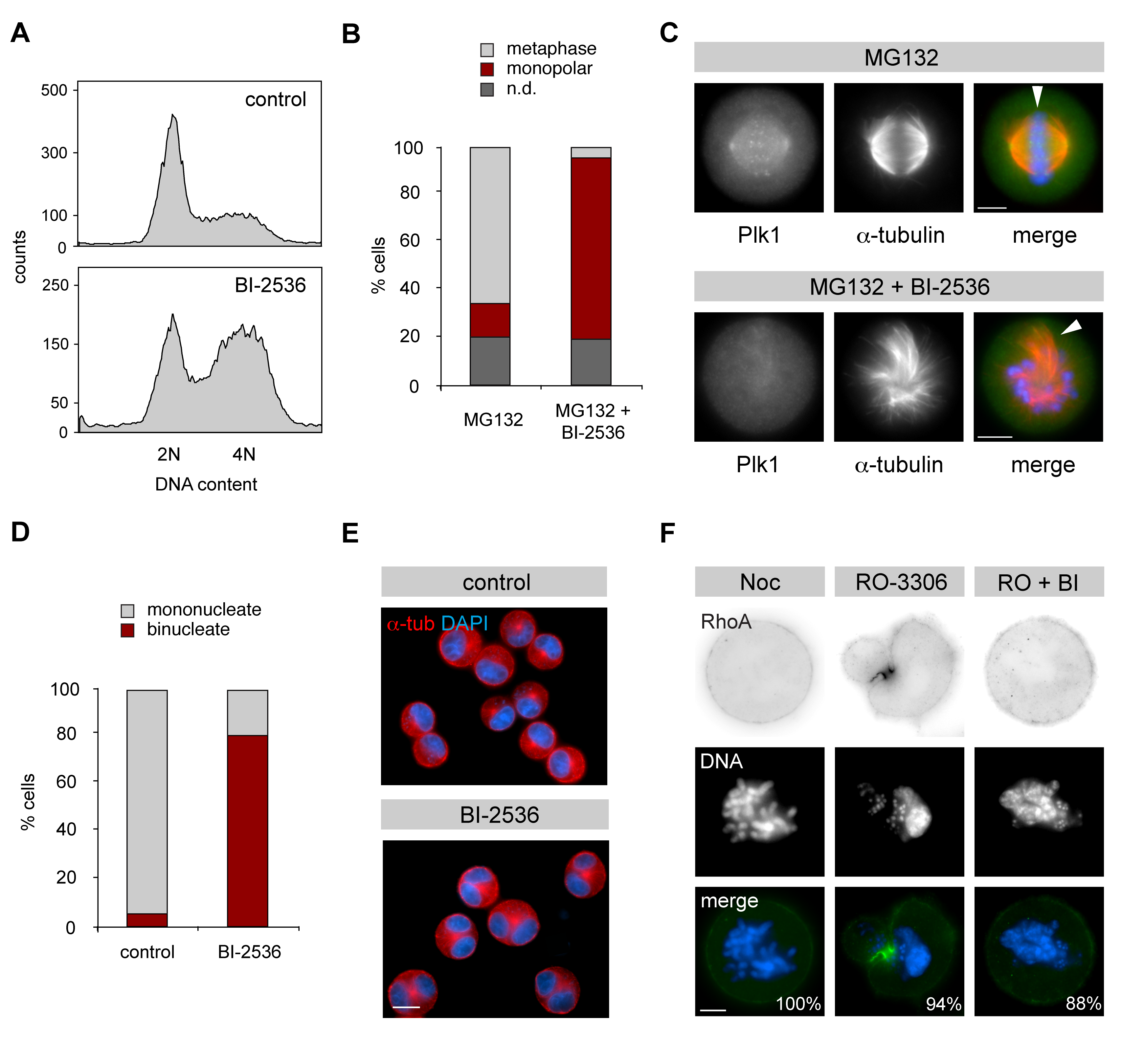

Supplement: Figure S7 — Effects of BI-2536 treatment on M phase progression in T. annulata -transformed cells. (A) Unsynchronized TaC12 cells (upper panel) or TaC12 cells cultured for 20 h in the presence of 100 nM BI-2536 (lower panel) were analyzed by flow cytometry. 2N, G1 phase; 4N, G2/M phase. (B) Metaphase-synchronized TaC12 cells were either kept in the presence of proteasomal inhibitor (MG132) or additionally treated with 100 nM BI-2536 (MG132 + BI-2536). Cells were harvested after 160 min and bi-/monopolar spindles were quantified by IFM; n.d. indicates cells that could not be classified; data represent 250 cells/sample. (C) Micrographs show representative T. annulata-infected cells with bipolar metaphase plate (MG132) or collapsed monopolar spindle (MG132 + BI-2536) that were quantified in (B). Cells were stained for Plk1 and α-tubulin and analyzed by IFM. DNA was stained with DAPI; arrowhead indicates the position of the parasite; scale bar represents 5 µm. (D) Metaphase-synchronized TaC12 cells synchronously released into anaphase and treated with DMSO (control) or 100 nM BI-2536 (BI-2536) at 15 min of release. Cells were analyzed after 4 h by IFM and the abundance of mononucleate and binucleate cells was determined for both samples; data represent 250 cells/sample. (E) Micrographs showing cells quantified in (D). Scale bar represents 10 µm. (F) S phase-synchronized TaC12 cells were released in the presence of nocodazole or 100 nM BI-2536 for 15 h and cells arrested in prometaphase were harvested from both cultures. Nocodazole-blocked prometaphase cells were either kept in the presence of the drug (Noc) or washed and treated with Cdk1 inhibitor for 30 min (RO-3306). Prometaphase cells obtained upon BI-2536 treatment were kept in the presence of 100 nM BI-2536 and additionally treated with Cdk1 inhibitor for 30 min (RO + BI). Cells were stained for RhoA and the occurrence of ectopic furrow ingression quantified (100 cells/sample) by IFM; DNA was stained with DAPI; scale bar repr [file pbio.1000499.s007.tif]

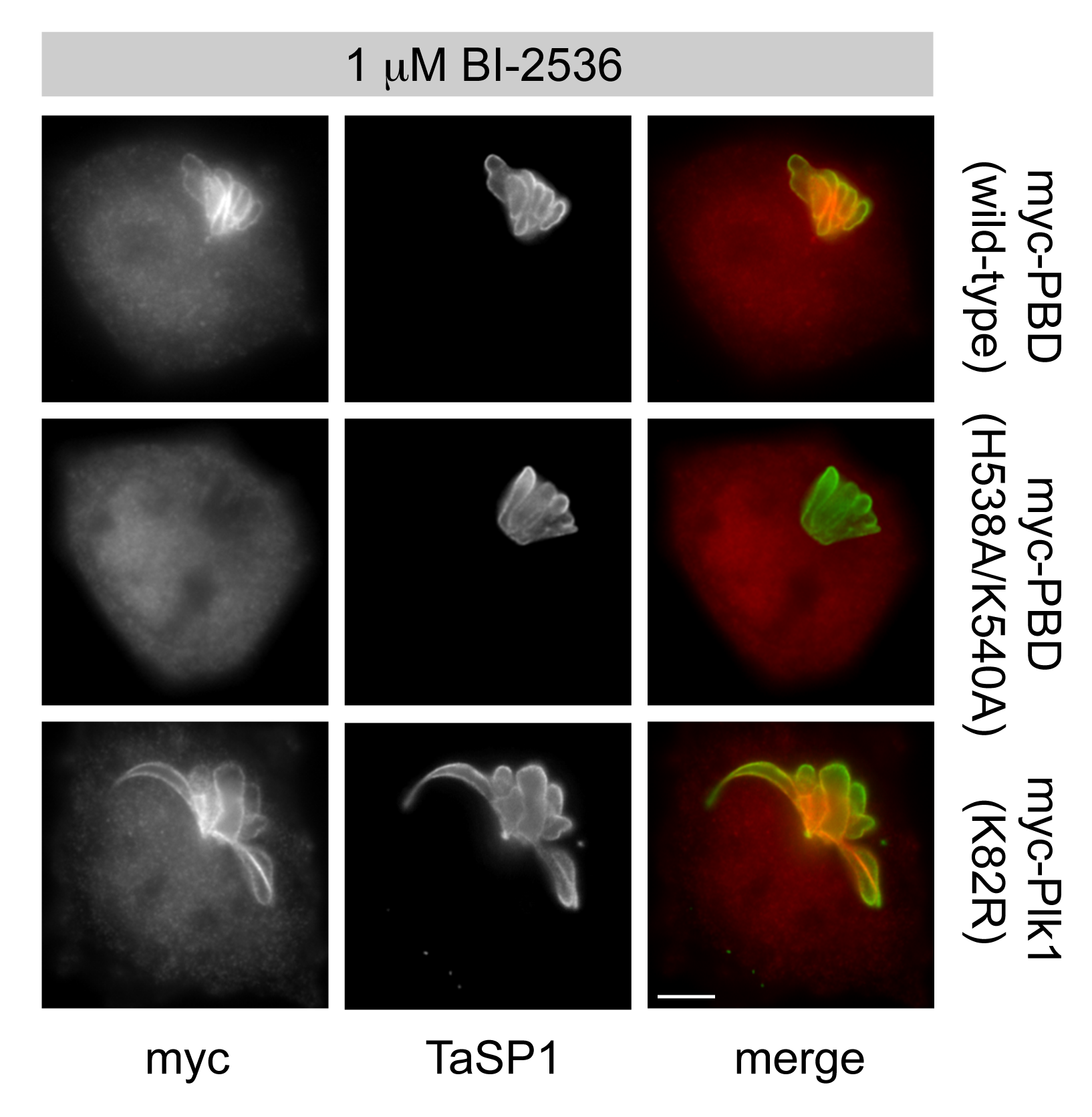

Supplement: Figure S8 — Ectopically expressed Plk1 PBD and catalytically inactive Plk1 can associate with the parasite surface in the presence of high doses of the Plk1 inhibitor BI-2536. T. annulata-infected cells were transfected with plasmids encoding myc-tagged versions of Polo-box domain (myc-PBD, wild type), H538A/K540A mutant PBD (myc-PBD H538A/K540A), or catalytically inactive Plk1 (myc-Plk1 K82R) and cultured in the presence or absence of BI-2536 at a concentration of 1 µM. Cells were analyzed by IFM using anti-myc and anti-TaSP1 antibodies. Scale bar represents 5 µm. (0.86 MB TIF) [file pbio.1000499.s008.tif]

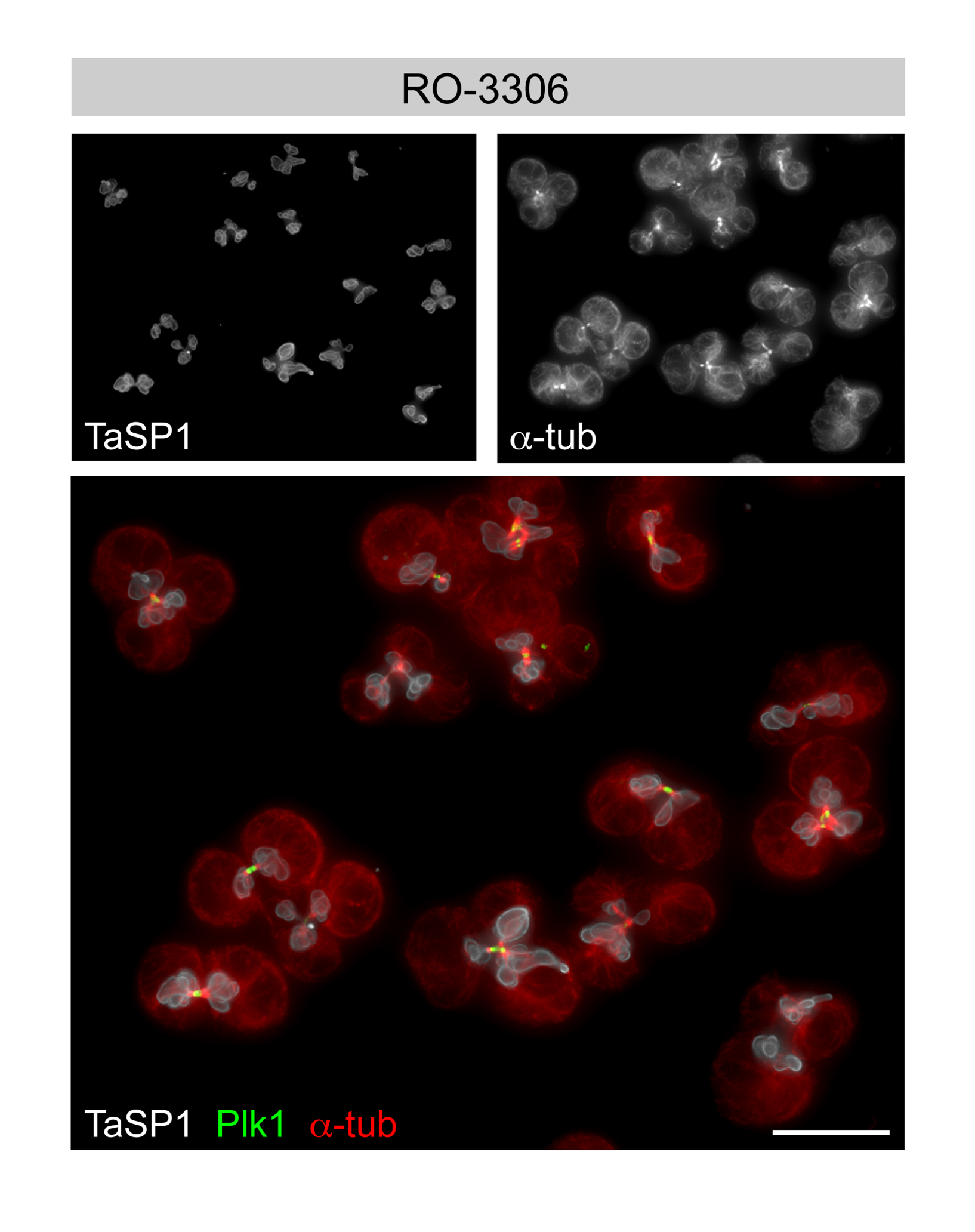

Supplement: Figure S9 — Cleavage furrow ingression occurs at sites where parasite-associated central spindle MTs assemble. T. annulata-transformed cells were synchronized in prometaphase, washed, and precocious anaphase and premature cytokinesis were induced by immediately blocking Cdk1 activity using the specific inhibitor RO-3306 (30 min). The formation of central spindles was monitored using anti-α-tubulin and anti-Plk1. The parasite was visualized using anti-TaSP1. Scale bar represents 20 µm. (1.07 MB TIF) [file pbio.1000499.s009.tif]

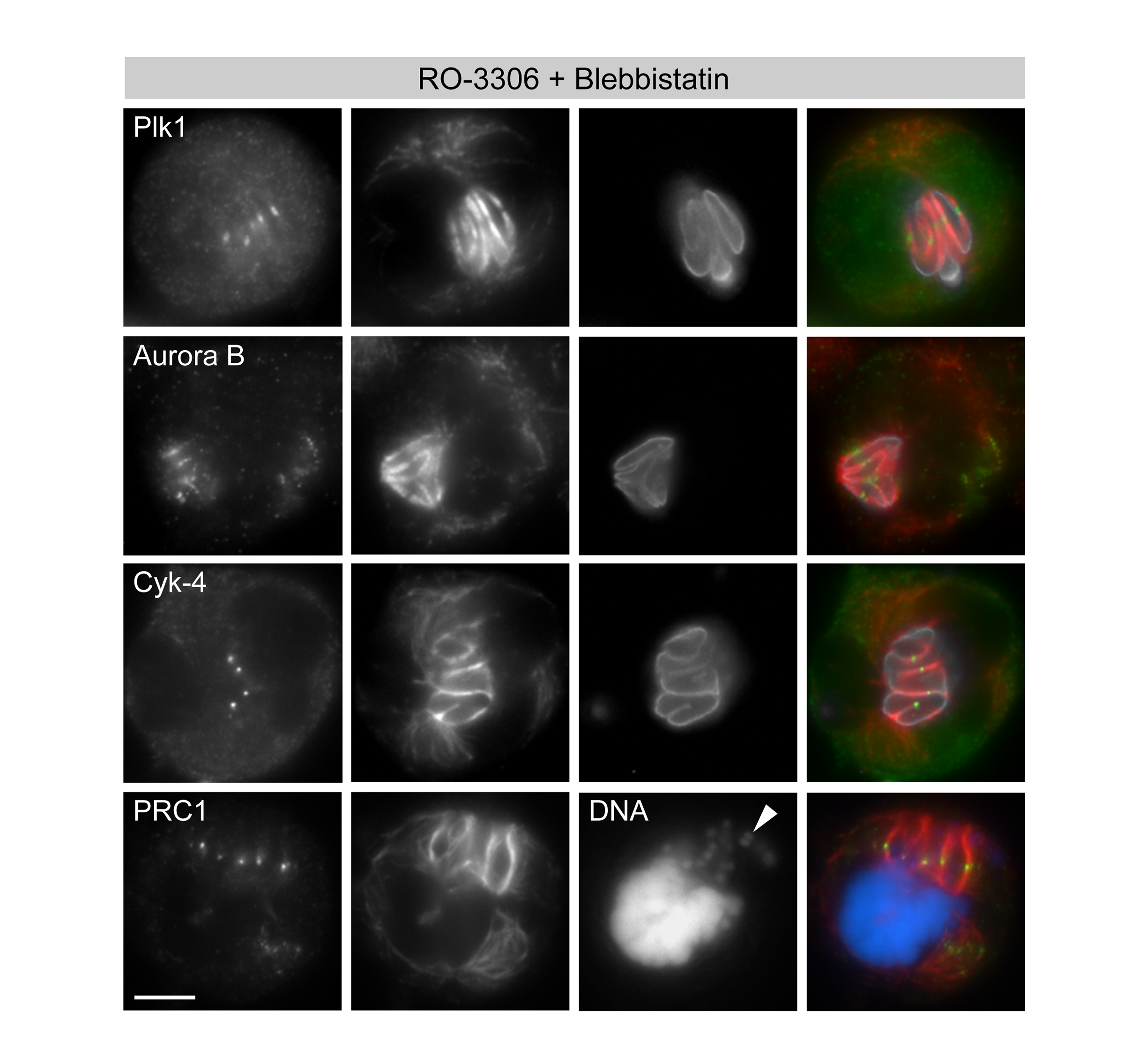

Supplement: Figure S10 — The Theileria schizont recruits central spindles to its surface. T. annulata-transformed cells were synchronized in prometaphase and precocious anaphase induced by blocking Cdk1 using the inhibitor RO-3306. Cleavage furrow contraction was prevented by treatment with the myosin II inhibitor blebbistatin. Parasite-associated central spindles were analyzed for the presence of central spindle-specific proteins such as Plk1, Aurora B, Cyk-4, and PRC1 as indicated. MTs were visualized using anti-α-tubulin, and the schizont was stained using anti-TaSP1; in the lower panels, DNA was stained with DAPI. Arrowhead indicates the position of the parasite. Scale bar represents 5 µm. (2.34 MB TIF) [file pbio.1000499.s010.tif]

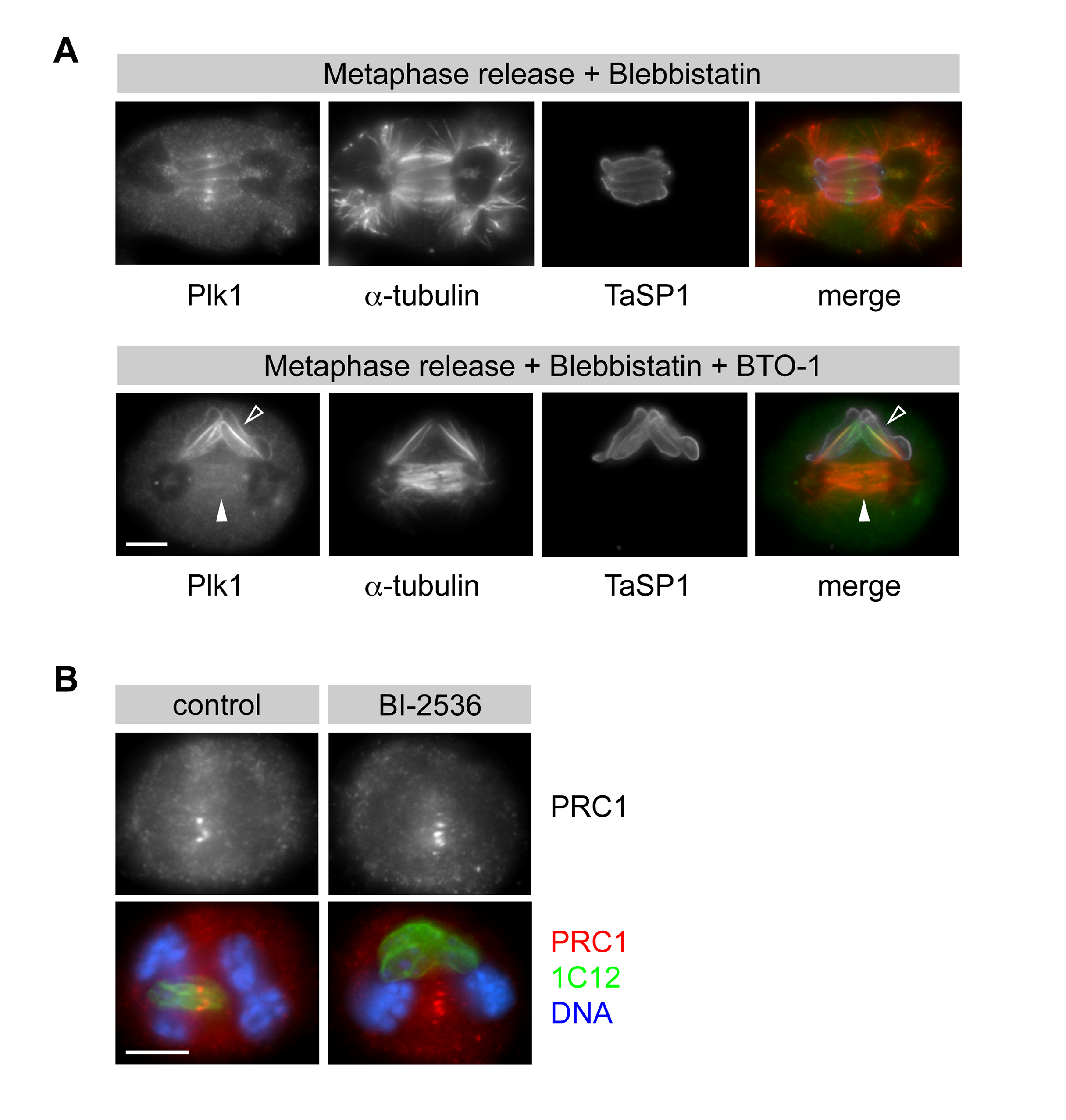

Supplement: Figure S11 — Recruitment of central spindle, but not astral, MTs to the parasite surface requires catalytically active Plk1. (A) T. annulata-transformed TaC12 cells were released for 80 min from metaphase arrest in the presence of the Plk1 inhibitor BTO-1 or control solvent. Cleavage furrow contraction was blocked by treatment with the myosin II inhibitor blebbistatin. Anti-α-tubulin was used to identify central spindles and parasite-associated MTs emanating from the spindle poles. Open arrowheads point at Plk1 binding to the schizont surface; closed arrowheads indicate central spindles lacking Plk1 in the central section. (B) Representative micrographs of IFM stainings that were used to quantitate the association of the parasite with central spindles in control cells and cells treated with Plk1 inhibitors (histogram Figure 9C). Cells were stained for the central spindle marker PRC1 and 1C12, a mouse monoclonal antibody that detects the surface of T. annulata schizonts; DNA was stained with DAPI. In cells treated with Plk1 inhibitor, the central spindle, detected by its marker PRC1, is not associated with the parasite. Scale bars represent 5 µm. (1.65 MB TIF) [file pbio.1000499.s011.tif]

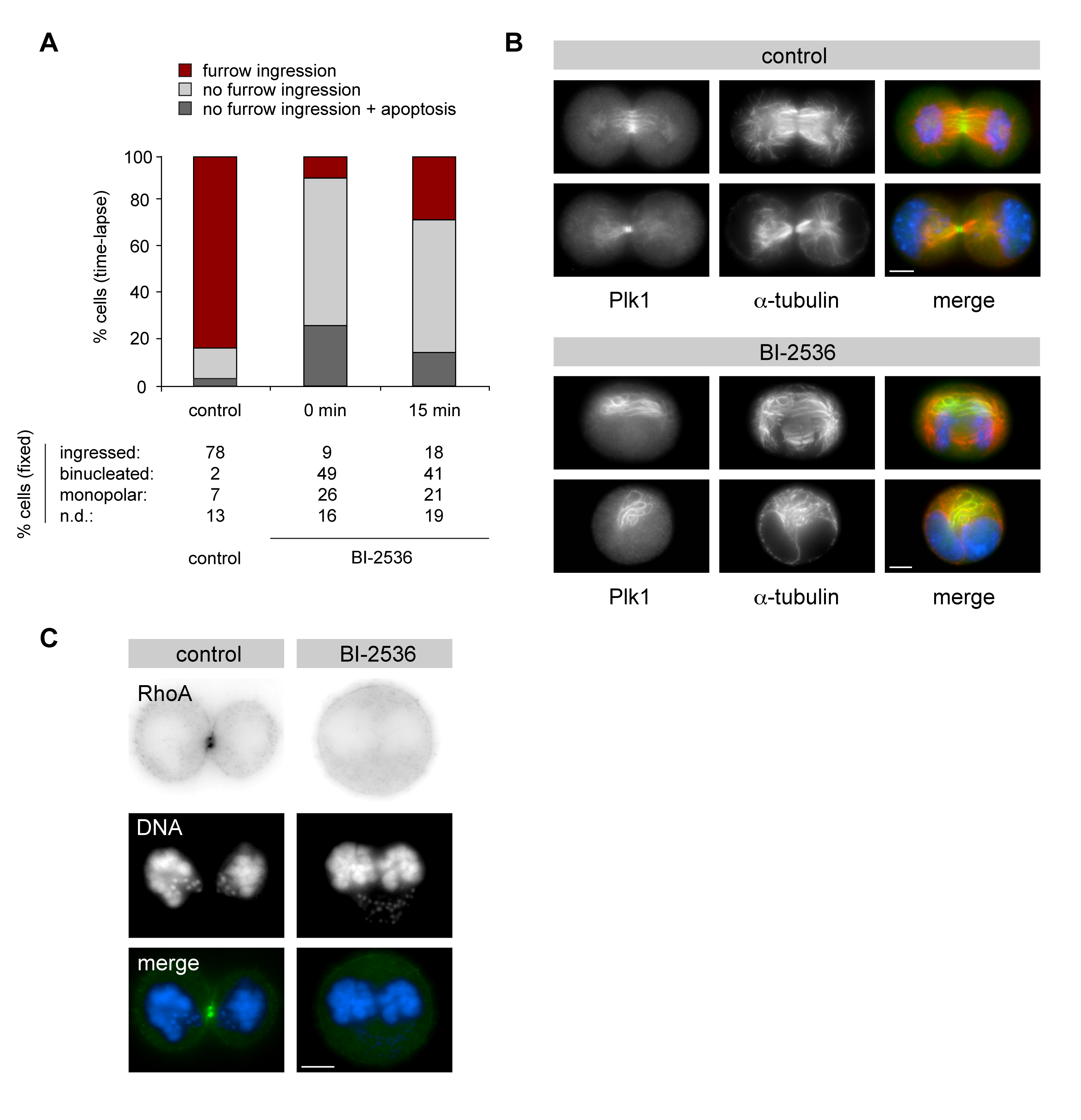

Supplement: Figure S12 — Effects of BI-2536 on furrow ingression in TaC12 cells. (A) T. annulata-infected cells were synchronized in metaphase using the proteasomal inhibitor MG132 and synchronously released into anaphase. Cells were treated with DMSO (control) or 100 nM BI-2536 (0 min) during MG132 washout; alternatively, cells were first exposed to 100 nM BI-2536, 15 min after MG132 washout (15 min). The histogram shows the abundance of furrow ingression in cells monitored by time-lapse imaging over a period of 4 h after MG132 washout. Additionally, cells were harvested at 80 min after MG132 washout, fixed, and analyzed by IFM (lower panel). Data are presented as the percentage of cells showing cleavage furrow ingression (ingressed), cells that lacked any furrow ingression (binucleation), or cells showing collapsed monoplar spindles (monopolar); n.d. denotes cells that could not be classified; data represent 200 cells/sample (time-lapse) or 300 cells/sample (fixed). (B) Micrographs of cells in anaphase or telophae obtained in (A) that were released in the presence of DMSO (control) or 100 nM BI-2536. Cells were stained for Plk1 as well as α-tubulin and analyzed by IFM; DNA was stained with DAPI. (C) Micrographs of representative cells obtained in (A). Cells were stained for RhoA and analyzed by IFM; DNA was stained with DAPI. Scale bars represent 5 µm. (1.87 MB TIF) [file pbio.1000499.s012.tif]
